# The *BDNF* Val66Met polymorphism is associated with the functional connectivity dynamics of pain modulatory systems in primary dysmenorrhea

**DOI:** 10.1038/srep23639

**Published:** 2016-03-24

**Authors:** Shyh-Yuh Wei, Hsiang-Tai Chao, Cheng-Hao Tu, Ming-Wei Lin, Wei-Chi Li, Intan Low, Horng-Der Shen, Li-Fen Chen, Jen-Chuen Hsieh

**Affiliations:** 1 Institute of Brain Science, School of Medicine, National Yang-Ming University, Taipei, Taiwan; 2 Department of Obstetrics and Gynecology, School of Medicine, National Yang-Ming University, Taipei, Taiwan; 3 Institute of Public Health, School of Medicine, National Yang-Ming University, Taipei, Taiwan; 4 Institute of Biomedical Informatics, School of Medicine, National Yang-Ming University, Taipei, Taiwan; 5 Integrated Brain Research Unit, Division of Clinical Research, Department of Medical Research, Taipei Veterans General Hospital, Taipei, Taiwan; 6 Department of Obstetrics and Gynecology, Taipei Veterans General Hospital, Taipei, Taiwan; 7 Laboratory of Microbiology, Division of Basic Research, Department of Medical Research, Taipei Veterans General Hospital, Taipei, Taiwan

## Abstract

Primary dysmenorrhea (PDM), menstrual pain without an organic cause, is a prevailing problem in women of reproductive age. We previously reported alterations of structure and functional connectivity (FC) in the periaqueductal gray (PAG) of PDM subjects. Given that the brain derived neurotrophic factor (BDNF) acts as a pain modulator within the PAG and the *BDNF* Val66Met polymorphism contributes towards susceptibility to PDM, the present study of imaging genetics set out to investigate the influence of, firstly, the *BDNF* Val66Met single nucleotide polymorphism and, secondly, the genotype-pain interplays on the descending pain modulatory systems in the context of PAG-seeded FC patterning. Fifty-six subjects with PDM and 60 controls participated in the current study of resting-state functional magnetic resonance imaging (fMRI) during the menstruation and peri-ovulatory phases; in parallel, blood samples were taken for genotyping. Our findings indicate that the *BDNF* Val66Met polymorphism is associated with the diverse functional expressions of the descending pain modulatory systems. Furthermore, PAG FC patterns in pain-free controls are altered in women with PDM in a genotype-specific manner. Such resilient brain dynamics may underpin the individual differences and shed light on the vulnerability for chronic pain disorders of PDM subjects.

Primary dysmenorrhea (PDM), menstrual pain without an organic cause, is the most prevalent menstrual complaint of women of reproductive age and is considered as a genuine chronic pain condition^1^. Between 40 to 90% of female adolescents have experienced PDM and 10–20% describe their suffering as so severe and distressing that it requires absence from school or work^1^. Notably, PDM subjects often display a higher prevalence of incidental brain findings, particularly of normal variants, when compared with control subjects^2^. Despite the unclear pathogenesis of PDM, myometrial hypercontractility and vasoconstriction are considered the two most widely accepted mechanisms^3^. It has recently been reported that a family history of dysmenorrhea strongly increases its risk by an odds ratio ranging between 3.8 and 20.7^4^.

We previously reported that long-term PDM is associated with brain-metabolism alterations and *state-* and *trait-related* structural changes^5^,^6^,^7^. *State-related* changes are menstrual pain-primed, whereas *trait-related* changes exist even without symptoms. Collectively, alterations of regional grey matter volume in PDM substantially involved brain regions of pain modulation systems (insula, medial prefrontal cortex [mPFC], periaqueductal gray [PAG], and etc.) that related either to the severity (insula and dorsal lateral prefrontal cortex [dlPFC]) or the duration (sensorimotor and posterior parietal cortex [PPC]) of the menstrual pain experience^6^. The PAG is a critical hub in the neuraxis of both the descending pain modulatory system and the ascending sensory system^8^. Notably, the PAG of the otherwise healthy PDM subjects demonstrated maladaptive functional hypo-connectivity (hypo-FC) with many key regions of the descending pain modulatory system (i.e., mPFC, PPC and dlPFC), whilst adaptive/reactive functional hyper-connectivity (hyper-FC) with the sensorimotor cortex^9^.

Brain-derived neurotrophic factor (BDNF) modulates the formation, maturation and plasticity of neuronal synapses^10^, including the central synapses, which form the pain circuits at both the spinal and supraspinal levels^11^; as a result BDNF plays a key role in central sensitization and chronic pain conditions^12^. The *BDNF* Val66Met polymorphism (rs6265) changes the BDNF protein sequence such that there is a valine (Val) to methionine (Met) substitution at codon 66^13^. It has been shown that this change reduces the activity-dependent secretion of BDNF^13^ and influences cortical pain processing^14^,^15^. An atypical cortical response to experimental pain has been associated with the Met allele in healthy subjects^14^. This effect may be mediated through the neurotrophin-induced neuroplasticity of various pain modulatory systems or via a direct BDNF neurotransmitter-like effect on the brainstem monoaminergic nuclei^14^, such as the raphe nuclei or the PAG. We have reported in our preliminary genetic association study that *BDNF* Val66Met polymorphism may contribute to the susceptibility of women to PDM^16^. In addition, presence of chronic pain (e.g., low back pain) may enhance genetic sensitivity to experimental pain when the Met allele is present^15^, indicating *BDNF* Val66Met polymorphism genotype-pain interplays and suggesting maladaptive neuroplasticity in Met allele carriers.

The PAG works in concert with the rostral ventromedial medulla via BDNF-containing projecting neurons, especially the ventrolateral subregion of the PAG^17^. Infusion of BDNF into the brainstem near the PAG causes an analgesic effect in rats^18^. PAG acts on the pain facilitation (ON cells) and pain inhibition (OFF cells) of the rostral ventromedial medulla modulating downstream transmission^19^. The dynamic shift in homeostatic regulation between pain facilitation and pain inhibition may either amplify or subdue central sensitization to pain^20^, which is a feature common to many chronic pain disorders, including irritable bowel syndrome, fibromyalgia, temporomandibular joint disease, chronic fatigue syndrome, chronic headache, and many others^21^. It is suggested that the maladaptive descending pain modulatory systems in young PDM subjects may underpin the central susceptibility to subsequent development of various functional disorders later in life^9^. However, how the *BDNF* Val66Met polymorphism attributes to the PDM vulnerability in the context of dynamic expressions of FC in the pain modulation systems remains elusive.

Given that the BDNF as a pain modulator within the PAG^11^,^17^ and the *BDNF* Val66Met polymorphism as a potential contributor for PDM susceptibility^16^, the present study used imaging genetics with the aim of investigating the influence of, firstly, the *BDNF* Val66Met single-nucleotide polymorphism and, secondly, the genotype-pain interplays related to PDMs in the context of PAG-seeded FC. We hypothesized that the menstrual pain experience of PDM could be represented by discrete PAG-seeded networks^9^, which in turn can be relevant to adaptive or maladaptive brain resilience among *BDNF* Val66Met polymorphism genotypes.

## Results

Since we have previously reported *BDNF* Met/Met homozygosity may be associated with an increased vulnerability of PDM^16^, thus we used the genotype information in the current study only for grouping purpose (Val/Val, Val/Met, and Met/Met) to elucidate the functional connectivity-genotype interplays in association with pain as a stressor. For the participant numbers and demographics, please see the Method section; for the detailed information of the genetic association with PDM, please refer to our published paper (Lee *et al*.^16^).

### Behavioral assessments

The PDM subjects exhibited lower mental and physical well-being (Table 1), while having higher scores for the Pain Catastrophizing Scale and Beck Depression Inventory (Supplementary Table S1) compared with the controls. However, there was no genotype difference associated with the Short-Form Health Survey, Pain Catastrophizing Scale or Beck Depression Inventory. All PDM subjects in this imaging genetics study had a long history of menstrual pain (mean ± SD = 9.48 ± 3.09 years), with the pain lasting approximately 1 to 3 days during one menstrual cycle (mean ± SD = 1.97 ± 0.75 days). The present and recalled pain experience, as assessed by scores on the McGill Pain Questionnaire, confirmed that PDM subjects experienced moderate to severe menstrual pain (Table 2). Thirty-four PDM subjects (60.7%) reported absences from school or work as a result of debilitating menstrual pain, and 23 PDM subjects (41.1%) used over-the-counter analgesics on occasion. Notably, only the Met/Met PDM groups showed a larger proportion of individuals using over-the-counter analgesics compared with no drug use (Table 2). Although the *p* value of chi-square test was not significant; nevertheless, the odds ratio was 4.286 (confidence interval: 1.058–17.363) for Met/Met PDM *vs*. Val/Met PDM, indicating a difference of pain management and efficacy between these genetic variants under similar recalled pain experience.

### Neuroimaging studies

#### PAG-seeded FC maps for the different genotypes within each group

**Figure 1:**
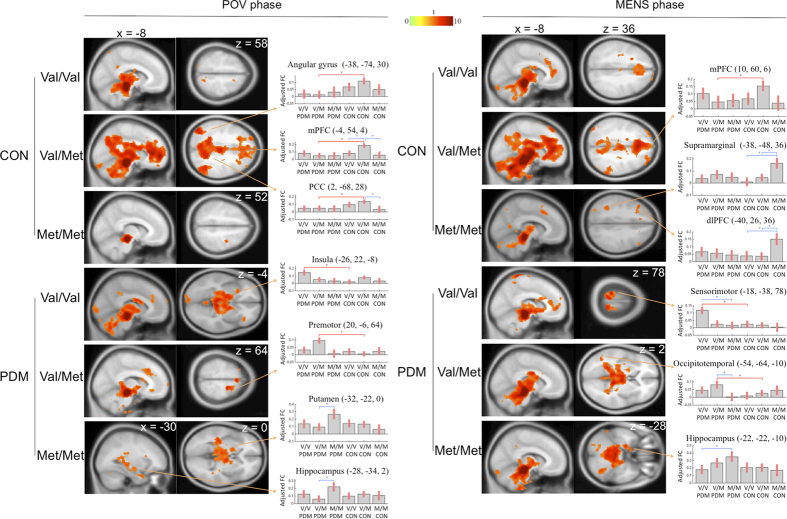
The Val66Met polymorphism of the *BDNF* is associated with the menstrual cycle variation in the functional expression of the descending pain modulatory system. Regions exhibit significant (FWE-corrected voxel level *p* < 0.05) resting-state functional connectivity with the PAG for between-group and/or between-genotype comparisons. Each region’s detail is listed in Tables 3 and 4 and Supplementary Table S3. The results are superimposed on the SPM T1 template, and the color bar represents *t*-scores. All figures adopt neurological orientation (left = left). The bar charts show the adjusted functional connectivity at the peak voxel of each region (coordinates in Montreal Neurological Institute space) for each genotype (V, valine; M, methionine) across the two groups (PDM, primary dysmenorrhea; CON, control) during the two phases (MENS, menstrual; POV, periovulatory). The error bar corresponds to a 90% confidence interval. *denote the contrasts that are significant in the between-group comparisons of each genotype (red) or between-genotype comparisons of each group (blue).

Among the control group, the different *BDNF* Val66Met polymorphism genotypes resulted in the engagement of different cortical modulatory pathways (Fig. 1). The Val/Val controls exhibited a PAG-PPC FC during the POV phase and exhibited a PAG-mPFC FC during the MENS phase. The Val/Met controls exhibited a FC between PAG and the default mode network (DMN; mPFC, precuneus/posterior cingulate cortex and angular gyrus) during both phases. The Met/Met controls exhibited both PAG-dlPFC and PAG-PPC FCs during the MENS phase.

However, the PAG of subjects with PDM exhibited FC with areas that are different from the controls. The Val/Val PDM subjects exhibited a PAG-insula FC during the POV phase and exhibited a PAG-sensorimotor FC during the MENS phase. The Val/Met PDM subjects exhibited a PAG-premotor cortex FC during the POV phase and PAG-occipitotemporal cortex FC during the MENS phase. The Met/Met PDM subjects exhibited PAG-hippocampus and PAG-pons FCs during both phases.

#### Between-genotype differences of each group

Among the control group, all the aforementioned PAG-seeded FCs were still significant after two between-genotype comparisons of the respective phases, except the Val/Val controls (Table 3). This may be because the Val/Val homozygous genotype is the wild type of *BDNF* Val66Met polymorphism, indicating that the Val to Met substitution results in additional FC.

During the MENS phase, among the PDM group, the PAG-sensorimotor FC was still significant after the comparison Val/Val > Met/Met, and the PAG-occipitotemporal cortex FC was still significant after the comparison Val/Met > Met/Met. During both phases, the Met/Met PDM subjects exhibited significantly higher PAG-hippocampus and PAG-pons FCs compared with both Val/Val and Val/Met PDM subjects.

#### Between-group differences for each genotype

Among the Val/Val subjects, the PAG-sensorimotor and PAG-insula FCs were still significant after the comparison PDM > CON during their respective phases (Table 4). Among the Val/Met subjects, the PAG-DMN FCs were still significant after the comparison CON > PDM during both phases, and the PAG-occipitotemporal cortex and PAG-premotor cortex FCs were still significant after the comparison PDM > CON of the respective phases. Among the Met/Met subjects, the PAG-pons FC was still significant after the comparison PDM > CON during the POV phase.

#### Correlation analyses between functional connectivity and the pain rating index of McGill Pain Questionnaire

The Val/Val PDM subjects exhibited *state*-related negative correlations between their present pain rating index and PAG-seeded FC in the mPFC, dlPFC, sensorimotor, secondary somatosensory cortex (S2), and middle temporal gyrus (Table 5). The Val/Val PDM subjects exhibited *trait*-related positive correlations between their recalled pain rating index and PAG-seeded FC in the DMN, while there were negative correlations in the ventrolateral prefrontal and orbitofrontal cortex. The Val/Met PDM subjects exhibited *trait*-related negative correlations between their recalled pain rating index and PAG-seeded FC in the sensorimotor cortex. Notably, only the Met/Met PDM subjects showed no correlation between their pain rating index and PAG-seeded FC.

## Discussion

Our findings indicate that the *BDNF* Val66Met polymorphism is associated with the diverse functional expressions of descending pain modulatory systems in the context of PAG-seeded FC. There are *BDNF* Val66Met polymorphism genotype-pain interplays within the systems that result in polyphyletic adaptive or maladaptive neuroplasticity that may be induced by long-term experience of menstrual pain. The Val/Val PDM subjects exhibit more adaptive neuroplasticity, whilst the Met/Met PDM subjects more maladaptive neuroplasticity. Such resilient brain dynamics may underpin the individual differences and shed light on the vulnerability for chronic pain disorders of PDM subjects.

Among the healthy subjects, individuals with different *BDNF* Val66Met polymorphism genotypes engaged different descending pain modulatory systems in terms of PAG-seeded FC variations. The Met/Met controls exhibited significant connectivity of the PAG-dlPFC and PAG-PPC FC, whereas the Val/Met controls exhibited significant connectivity of the PAG-mPFC FC (Fig. 1). These FCs are all associated with the attentional modulation of pain^22^,^23^. In addition, white matter integrity within and between these regions is known to be critically linked with an individual’s ability to control pain^24^. It is tempting to reason that these inherent differences in the genetic status of the subjects (their genetic predisposition) may contribute to individual differences in the context of brain resilience of FCs and its relationship with pain modulation and behavior. This view is corroborated by a growing body of evidence wherein individuals with different *BDNF* Val66Met polymorphism genotypes exhibit differences in brain plasticity expressions as induced by motor learning^25^, brain stimulation^26^, experimental pain stimulation^14^, and even when carrying out a simple motor task^27^. Furthermore, results from the present study pinpoint a large amount of variability in the FC amongst all *BDNF* Val66Met polymorphisms (genetic influences) and menstrual phases, indicating *BDNF* Val66Met polymorphism genotype-pain interplays. We argue that using “Met allele carriers” (collating Val/Met heterozygotes and Met/Met homozygotes as one single genotype group) to wrap up the data^14^,^15^ seems to be an oversimplified approach.

PDM subjects were found in this study to exhibit altered PAG-seeded FCs as compared to the healthy controls, and each *BDNF* Val66Met polymorphism genotype demonstrated a unique circuitry with significant differences between the groups and /or the genotypes (Tables 3 and 4). It has been reported that the *BDNF* Val66Met polymorphism plays a significant role in shaping human brain plasticity and that this occurs in an induction mechanism-specific manner^28^,^29^. The effect of the *BDNF* Val66Met polymorphism genotype-pain interplays on the descending pain modulatory systems thus result in polyphyletic adaptive or maladaptive neuroplasticity when induced by a long-term experience of menstrual pain. This *BDNF* Val66Met polymorphism genotype-pain interplay has also been reported among low back pain patients^15^ and it would seem that experiencing chronic pain may enhance a genetic (the Met allele) sensitivity to experimental pain. The mechanisms underlying such neuroplasticity remain unknown; the activity-dependent BDNF release mechanisms may be involved because stress evokes adaptive changes that might be linked to an increased expression of the *BDNF* gene in the PAG^30^, but *BDNF* Val66Met polymorphism reduces the activity-dependent secretion of BDNF^13^.

The Val/Val PDM subjects exhibited PAG-insula FC during the POV phase and PAG-sensorimotor FC during the MENS phase, which indicate adaptive neuroplasticity in the descending pain modulatory systems. The descending pathways from the insula to the PAG via amygdala provide the attentional modulation of pain^31^. In addition, evidence exists for the analgesic effects of primary motor cortex stimulation, possibly through the corticothalamic projections of motor cortex to the PAG^32^; therefore, this PAG-sensorimotor FC may imply a spontaneous engagement of a top-down modulation pathway that involves the motor cortex and pyramidal tract^9^. Our reasoning is supported by the *state*-related negative correlation between the PAG-sensorimotor FC and their present pain rating index: the higher the functional coupling, the lower the rating. Furthermore, the PAG-mPFC, PAG-dlPFC, and PAG-S2 FCs (all associated with the cortical modulation of pain^22^,^31^) are all negatively correlated with their present pain rating index, suggesting adaptive neuroplasticity in the Val/Val PDM subjects (Table 5).

Among three genetic subgroups with PDM, only the Met/Met PDM subjects exhibited stationary PAG-seeded FC throughout their menstrual cycle (a loss of condition-dependent dynamics), which suggests a maladaptive neuroplasticity in descending pain modulatory systems that is associated with less resilience as part of pain modulation. The stationary PAG-hippocampus and PAG-pons FCs may affect the formation of the PAG FC in association with the relevant brain regions involved in descending pain modulation, namely the frontal or parietal cortex. It is noteworthy that only the Met/Met PDM subjects exhibited no correlation between their pain rating index and PAG-seeded FC (Table 5). Such “stuck in a rut” phenomenon that shows little flexibility in terms of interactions with other networks has been reported by a recent FC study on temporomandibular disorder patients; it reported that an overtly enhanced FC within DMN system may impede the formation of FC with PAG for effective pain modulation^33^.

Moreover, only the Met/Met PDM subjects exhibited significant PAG FC with limbic structures (hippocampus and basal ganglia), indicating a possible pain chronicity of the studied Met/Met PDM subjects^34^. It is emphasized that the shift in the brain’s representation of pain from sensory regions, namely the insula and sensorimotor regions, to emotional and limbic structures is a pivotal neuromarker when pain moves towards a chronic state^34^. The study also indicates that the connectivity of hippocampus is intensely engaged during the transition to chronicity, and their findings in chronic state exhibit a midbrain region (encompassing the PAG) that may be related to prediction of chronification. Therefore, the Val/Val homozygosity may be protective because it mainly engages sensory regions of the pain matrix, while Met/Met homozygosity might render individuals vulnerable to menstrual pain by engaging limbic structures as well as contributing to the future development of functional chronic pain^9^,^16^.

The Val/Met PDM subjects, similar to the Val/Val PDM subjects, exhibited correlations between their pain rating index and PAG-sensorimotor FC. However, the regions surviving between-group and between-genotype comparisons among the Val/Met PDM subjects (the premotor and occipitotemporal cortex) are not the classical neural substrates of the descending pain modulatory systems. Whether Val/Met PDM females can be associated with adaptive neuroplasticity requires further investigation since neither dominant/recessive nor additive effect can fully explicate the data as disclosed. Further investigations are mandatory to address the issues. Nevertheless, the findings are in line with the existing literatures, which collectively posit that Val/Met subjects show greater similarity to Val/Val subjects^13^,^35^. Furthermore, the composite nature of Val/Met heterozygosis may potentially result in greater dynamic complexity and resilience of the brain system in a region specific way.

Brain imaging can be more sensitive than behavioral measurements, i.e., the neuroimaging findings can be sub-clinical or pre-clinical without conspicuous behavioral manifestation^36^. The gene-brain relationship is clearly not just a one-to-one correspondence with respect to behavioral expression because the penetrance of genes can be greater at the level of brain biology than at the level of behavior^37^,^38^. In fact, many imaging genetics studies have reported subclinical differences in brain signatures among the *BDNF* Val66Met polymorphism genotypes without there being any noticeable behavioral differences among the groups^14^,^15^,^25^,^27^; one possibility is that certain compensatory mechanisms in terms of brain plasticity may occur that, at least partially, may compensate for the genetic differences^27^,^39^. Collectively, these data present genetic complexity for functional and structural constitutions of brain. Therefore, the PDM subjects with different genotypes may show different FC dynamics, but without conspicuous behavioral differences in their pain experience. It should be born in mind that an attribution such as vulnerability to an illness may stem not only from a single single-nucleotide polymorphism effect, but also the interactions and contributions of multiple genes^40^. Such gene–gene interactions need not be simple additive; rather, the function of one gene on the brain may be dependent on the prior function of one or more other genes, a phenomenon known as epistasis^41^. Such genes interactome in the human brain has been reported in Schizophrenia^42^.

## Conclusions

The *BDNF* Val66Met polymorphism is involved in the diverse functional expressions of the pain modulatory systems in terms of PAG-FCs. When confronted with the repeated stress of menstrual pain, the functional dynamics of the system undergo further differential changes that vary with the *BDNF* Val66Met genotype of the individual. These genetic factors that affect brain resilience might contribute to individual differences when experiencing pain and influence the coping mechanisms of the PDM subjects as well as perhaps affecting their vulnerability in the future for the development of other chronic pain disorders. Whether the observed *BDNF*-genotype predilection of functional dynamics of pain modulatory systems is general to other chronic pain disorders remains open for further studies.

## Materials and Methods

### Subjects

The subjects of this study were a subset of the participants (smaller in sample size) from our previously published genetic association and behavioral studies of PDM who had completed the whole study protocols and were eligible for this imaging genetics study^16^. The genotype information was only used for grouping purpose in the current study. In brief, fifty-six otherwise healthy PDM subjects (Val/Val *n* = 19, Val/Met *n* = 20, Met/Met *n* = 17) and 60 healthy female controls (Val/Val *n* = 19, Val/Met *n* = 29, Met/Met *n* = 12) who were of the same ethnicity (Chinese) participated in the present study. No significant between-group differences were detected for the demographic data (*p* > 0.05; Table 1) and the *BDNF* Val66Met polymorphism (*p* = 0.304). The *BDNF* Val66Met-genotype distribution did not deviate from the Hardy–Weinberg equilibrium (*p* = 0.11) in this subset of subjects, implicating conformity with the population distribution.

All participants, who were recruited from Internet advertisements, were screened using telephone and in-person structured interviews regardless of case or control status. All participants were double screened and diagnosed at a gynecology clinic by a gynecologist (H.T.C.). The inclusion criteria for the PDM group were the following: 1) a regular menstrual cycle of approximately 27–32 days; 2) a history of PDM longer than 6 months; 3) an averaged menstrual pain under regular treatment with a rating that was higher than 4 on a verbal numerical scale (VNS, 0 = not at all, 10 = the worst imaginable pain) over the last 6 months; and 4) right-handedness, as confirmed by the Edinburgh Handedness Inventory^43^. The inclusion criteria for the healthy female controls were similar to those for the PDM group, except that the controls had no pain whatsoever during menses (VNS = 0). The exclusion criteria for all the participants were as follows: 1) using oral contraceptives, hormonal supplements, Chinese medicine, or any centrally acting medication (e.g., opioid, anti-epileptics) within 6 months prior to the study; 2) pathological pituitary gland disease; 3) organic pelvic disease; 4) any psychiatric or neurological disorders, particularly premenstrual dysphoric disorder; 5) any head injury with loss of consciousness; 6) immediate plans for pregnancy or a positive pregnancy test; 7) a history of childbirth; and 8) having a metal/pacemaker implant, claustrophobia, or any contraindications in relation to MRI. No analgesics had been taken by the subjects within 24 hours before the study. All PDM subjects received pelvic ultrasonography to exclude secondary dysmenorrhea caused by an organic pelvic disease such as endometriosis or adenomyosis. The study was conducted in accordance with the Declaration of Helsinki and was approved by the Institutional Review Board of Taipei Veterans General Hospital. All participants gave their written informed consent.

### Experimental design

MRI scans were individually scheduled according to each subject’s first day of menstruation. At two time points during the menstrual cycle: the menstruation phase (MENS phase, days 1–3 of the menstrual cycle) and the periovulatory phase (POV phase, days 12–16 of the menstrual cycle) blood samples were taken and subjected to a gonadal hormone assay and then MRI images (T1 and resting-state fMRI images) were obtained for the functional dynamics of the PAG-seeded FCs across fluctuating pain (off during POV, on during MENS).

### Psychological and quality-of-life assessments of the subjects’ pain experience

During the initial examination all participants in the two groups completed the Short-Form Health Survey (SF-36)^44^ to assess their quality of life. The PDM subjects completed the McGill Pain Questionnaire during the initial examination and during the MENS phase in order to assess their recalled overall and present experiences of menstrual pain, respectively. All participants in the two groups completed the Pain Catastrophizing Scale^45^ and Beck Depression Inventory^46^ during the MENS and POV phases in order to assess their pain-maladaptive psychological status and depressive symptoms, respectively.

### Genotyping

Blood samples for genotyping were obtained during the initial examination. Whole blood was collected in 4 mL EDTA tubes and stored at 4 °C in a fridge. DNA extraction was subsequently performed using the Puregene kit by following the manufacturer’s guidelines (Gentra Systems, Minneapolis, MN). Commercial TaqMan single-nucleotide polymorphism assays (Applied Biosystems, Foster City, CA) were used for genotyping. The polymerase chain reaction amplification was conducted in a total volume of 10 μL using the following amplification protocol: 50 °C for 2 min, 95 °C for 10 min, and 40 cycles of 92 °C for 15 sec and 60 °C for 1 min. Fluorescence measurements were performed using the ABI HT7900 (Applied Biosystems, Foster City, CA), and allele calling was performed by the SDS 2.2 software package (Applied Biosystems). Genotypes were independently assigned to the subjects by two technicians who were blinded to the subjects’ personal information.

### Serum gonadal hormone measurements

The sera extracted from the blood samples drawn during the MENS and POV phases were stored for batch analysis using commercialized assays (UniCel DxC 800 Synchron Clinical Systems, Beckman Coulter, Inc., Brea, CA). The total serum concentrations were assayed using a chemiluminescence immunoassay technique for estradiol and progesterone and a radioimmunoassay technique for testosterone. There were phase differences for estradiol and testosterone, genotype differences for testosterone, and an interaction between phase and genotype for testosterone. No other main effects or interactions were noted (Supplementary Table S2). As gonadal hormones may affect the resting-state FC^47^,^48^,^49^, the hormone fluctuations were regressed out as covariates of non-interest during the subsequent image processing.

### Image acquisition

Resting-state functional MRI images were acquired using a 3.0 Tesla MRI scanner (Magnetom Trio Tim, Siemens, Erlangen, Germany) with a 12-channel head coil at National Yang-Ming University. High-resolution T1-weighted 3-dimensional structural images using a magnetization-prepared rapid-acquired gradient echo sequence (MPRAGE; [TR]/[TE] = 2530 ms/3.03 ms, flip angle = 70°, field-of-view = 224 × 256 × 192 mm^3^, in-plane matrix size = 224 × 256 × 192, in-plane resolution = 1 mm) and T2*-weighted gradient echo sequence ([TR]/[TE] = 2500 ms/30 ms, flip angle = 90°, field-of-view = 220 × 220 × 136 mm^3^, in-plane matrix size = 64 × 64 × 40, in-plane resolution = 3.4 mm [round-out], and 204 volumes per run) were conducted to obtain high-resolution anatomical T1 images and functional MRI images^9^. The first 4 functional scans of each resting-state functional MRI series were discarded for signal saturation and magnetic field stabilization. The participants remained awake during the scan (eyes open, heads still but relaxed, without thinking about anything in particular). Head cushions and earplugs were provided to reduce head motion and noise, respectively.

### Image preprocessing

Preprocessing was performed using the DPARSF toolbox (State Key Laboratory of Cognitive Neuroscience and Learning, Beijing Normal University, China) with Statistical Parametrical Mapping 8 (SPM8, Wellcome Trust Centre for Neuroimaging, London, http://www.fil.ion.ucl.ac.uk/spm) in Matlab. All functional images were subjected to slice timing, realignment for head-motion correction, co-registration against each individual’s anatomical image as well as normalization against the Montreal Neurological Institute (MNI-152) template. Subjects having head motion of any volume more than 2 mm or 2° were excluded from further processing^50^. The images were re-sampled to an isotropic 2 mm^3^ voxel size during the normalization step and then spatially smoothed using a 3D Gaussian kernel of 8 mm full-width at half-maximum. Linear trends were then removed from the resulting time series, and the time series was temporally band-pass filtered (0.01–0.08 Hz) in order to extract the low-frequency oscillations associated with spontaneous neuronal activity^51^.

### Removal of physiological and scanner-related noise

The averaged time courses of the following nuisance variables or confounding artifacts were regressed out: 1) the six head-movement parameters computed based on rigid body translation and rotation during the realignment in SPM8, 2) the global mean signal (global signal regression), 3) the mean signal within the lateral ventricles, and 4) the mean signal within a deep white matter region (centrum ovale). The cerebrospinal fluid and the white-matter signals are thought to reflect fluctuations in non-specific regional correlations. We performed global signal regression because it can maximize the spatial specificity of positive resting-state correlations^52^, improve correspondence to anatomy^53^ and to electrophysiology^54^. The neuroscientific interpretation of anti-correlation has been challenged^55^, and global signal regression may cause a negative shift in the distribution of correlations^53^,^56^,^57^; therefore, we implemented a mask and addressed positive connectivity only in order to remove distortion after global signal regression^9^,^58^.

### Definition of PAG seed and PAG-seeded FC maps

The PAG seed (3-mm radius), centered at MNI coordinates [−4, −26, −14], was identified based on the published literature^59^,^60^. The mean time-series activity in the seed region of each subject was extracted. PAG-seeded FC maps were then generated. Each individual-level FC map obtained was then converted into a z-map using Fisher’s *r*-to-*z* transformation for second-level group analyses^50^.

### Statistical analyses

#### Demographic information and psychophysiological measurements

SPSS Statistics 20.0 (SPSS Inc., Chicago, IL) was used for all of these analyses. The results were considered significant at *p* < 0.05 (two-tailed). The Hardy-Weinberg equilibrium of the *BDNF* genotype distribution and the menstrual pain experience of PDM (absences from school or work, analgesics taken) were examined using the chi-square test. A one-way analysis of variance (ANOVA) of the pain history and pain rating index of McGill Pain Questionnaire was conducted to assess the main effect of the *BDNF* genotype (Met/Met homozygotes *vs*. Val/Val homozygotes *vs*. Val/Met heterozygotes). A two-way ANOVA of the demographic characteristics, Edinburgh Handedness Inventory scores and Short-Form Health Survey scores was conducted to assess the main effects of group (PDM *vs*. CON) and *BDNF* genotype (Met/Met homozygotes *vs*. Val/Val homozygotes *vs*. Val/Met heterozygotes), as well as the interaction between them. A *post-hoc* two-sample *t*-test was performed whenever the *BDNF* genotype or the interaction was significant. To assess the serum hormone levels, Pain Catastrophizing Scale and Beck Depression Inventory scores during the two phases, a general linear model with a repeated-measures design was applied to examine the possible effects of group (PDM *vs*. CON), *BDNF* genotype (Met/Met homozygotes *vs*. Val/Val homozygotes *vs*. Val/Met heterozygotes) and menstrual cycle phase (MENS *vs*. POV), as well as the interactions between these factors.

#### Image analysis

For each phase, a mixed-effects model of factorial design (2 factors: genotype and group) was employed to analyze the FC maps using SPM 8 (Wellcome Trust Centre for Neuroimaging, London, http://www.fil.ion.ucl.ac.uk/spm). Gonadal hormones were regressed out as covariates of non-interest. The connectivity maps (Fig. 1) for the different genotypes in each group were statistically examined using the one-tailed one-sample *t* test. Significance was thresholded at the FWE-corrected voxel level *p* = 0.05. Statistical maps (see Supplementary Fig. S1 online) were computed to identify changes in PAG-seeded FC for the following contrasts: 1) between-group comparisons for each genotype and 2) between-*BDNF* genotype comparisons for each group. Significance was thresholded at the uncorrected voxel level *p* = 0.005, followed by the FDR-corrected cluster level *p* = 0.05.

In PDM group, a mixed-effects model of factorial design (1 factor: genotype) was conducted to determine the correlation between the present or recalled overall experience of menstrual pain and the *state* or *trait* PAG-seeded FC for each *BDNF* genotype, respectively. We first entered the demeaned (in SPM) pain rating index from the present or recalled McGill Pain Questionnaire as a regressor to identify brain regions with either positive or negative correlations with PAG-seeded FC during the MENS or POV phase in each *BDNF* genotype. Gonadal hormones were regressed out as covariates of non-interest. Correlations during the MENS phase (painful stage) were regarded as *state* relationships, whereas correlations during the POV phase (pain-free stage) or throughout the menstrual cycle were regarded as *trait* relationships. Significance was thresholded at the uncorrected voxel level *p* = 0.005, followed by the FDR-corrected cluster level *p* = 0.05.

Since the SPM would report peak coordinates as identified within a confluent cluster, there can be multiples peaks that sit on different brain regions/areas (e.g., Brodmann area). We would report one representing peak (the maximum) for each region/area, respectively (e.g., in Table 3, hippocampus, putamen and thalamus belong to a same cluster).

## Additional Information

**How to cite this article**: Wei, S.-Y. *et al*. The *BDNF* Val66Met polymorphism is associated with the functional connectivity dynamics of pain modulatory systems in primary dysmenorrhea. *Sci. Rep.* **6**, 23639; doi: 10.1038/srep23639 (2016).

## Supplementary Material

Supplementary Information

## Figures and Tables

**Table 1:** Demographic data and baseline information.

	PDM (*n* = 56)	CON (*n* = 60)	*p* Value		
			Main effect of group	Main effect of genotype	Interaction
Age, year	23.1 (2.33)	23.9 (2.42)	0.060	0.264	0.058
Met/Met	22.3 (1.45)	23.8 (1.85)			
Val/Met	24.3 (2.89)	23.7 (2.44)			
Val/Val	22.7 (2.00)	24.3 (2.77)			
Years of menstruating	11.3 (2.71)	11.5 (2.72)	0.540	0.451	0.078
Met/Met	10.7 (2.12)	11.4 (2.64)			
Val/Met	12.4 (3.46)	11.2 (2.44)			
Val/Val	10.6 (1.89)	12.0 (3.20)			
Days of one menstrual cycle	29.4 (1.42)	29.5 (1.14)	0.412	0.129	0.930
Met/Met	29.8 (0.81)	29.9 (1.24)			
Val/Met	29.2 (1.38)	29.4 (1.16)			
Val/Val	29.2 (1.82)	29.5 (1.04)			
Edinburgh Handedness Inventory	81.8 (17.78)	80.3 (19.39)	0.643	0.850	0.257
Met/Met	78.8 (19.33)	82.6 (19.55)			
Val/Met	81.5 (19.80)	82.9 (19.61)			
Val/Val	84.9 (14.27)	74.7 (18.85)			
Short Form Health Survey (SF-36)*					
Mental component summary	46.3 (8.38)	53.4 (5.64)	0.000	0.365	0.194
Met/Met	46.4 (9.51)	50.5 (7.53)			
Val/Met	46.7 (8.02)	52.9 (5.00)			
Val/Val	45.8 (8.11)	56.2 (3.89)			
Physical component summary	46.2 (10.14)	54.4 (5.09)	0.000	0.327	0.432
Met/Met	47.1 (11.34)	51.9 (6.19)			
Val/Met	46.6 (10.62)	56.3 (4.16)			
Val/Val	45.0 (8.85)	52.9 (4.64)			

^*^Three control subjects did not complete the Short Form Health Survey and were excluded from these calculations.

PDM, primary dysmenorrhea; CON, control; Val, valine; Met, methionine. The data are presented as the means (SD).

**Table 2:** *BDNF* rs6265 genotype effects on the menstrual pain experience of PDM.

	Met/Met (*n* = 17)	Val/Met (*n* = 20)	Val/Val (*n* = 19)	Total (*n* = 56)	*p* Value
**Absenteeism (%)**	11 (64.7%)	12 (60%)	11 (57.9%)	34 (60.7%)	0.942
No absenteeism (%)	6 (35.3%)	8 (40%)	8 (42.1%)	20 (39.3%)	
**Drug taken (%)**	10 (58.8%)	5 (25%)	8 (42.1%)	23 (41.1%)	0.113
No drug taken (%)	7 (41.2%)	15 (75%)	11 (57.9%)	33 (58.9%)	
**Pain history, year (SD)**	8.9 (2.21)	10.8 (3.28)	8.7 (3.27)	9.5 (3.09)	0.069
**Recalled PRI scores (SD)**	34.7 (13.19)	36.0 (12.80)	34.2 (15.61)	35.0 (13.69)	0.923
Present PRI scores* **(SD)**	34.7 (13.65)	29.1 (10.03)	29.9 (13.29)	31.2 (12.35)	0.358

^*^Three PDM subjects did not complete the McGill Pain Questionnaire and were excluded from this calculation.

*BDNF*, brain-derived neurotrophic factor; PDM, primary dysmenorrhea; PRI, pain rating index; Val, valine; Met, methionine.

**Table 3:** Peak MNI coordinates of the regions exhibiting significant resting-state functional connectivity with the PAG for the between-genotype differences of each group.

Group	Contrast of genotype	Phase	Region, Laterality	BA	Cluster	*t* Score	Peak coordinate		
							*x*	*y*	*z*
CON	Val/Met > Val/Val	POV	PFC, M	10	2492	4.18	−6	52	0
	Val/Met > Met/Met	POV	PFC, M	10	792	3.84	−4	54	4
		POV	PCC/precuneus, M	31	276	3.14	2	−68	28
	Met/Met > Val/Val	MENS	Supramrginal, L	40	386	4.59	−38	−48	36
		MENS	dlPFC, L	9	370	3.68	−40	26	36
	Met/Met > Val/Met	POV	Cuneus, L	18	482	4.35	−36	−86	−6
		POV	Cuneus, R	19	460	4.45	38	−76	−8
		POV	Premotor cortex, R	6	307	5.69	36	−8	52
		POV	SMA, M	6	286	4.51	2	−16	60
	Met/Met > Val/Met	MENS	Supramrginal, L	40	297	4.08	−40	−48	40
		MENS	dlPFC, L	9	316	4.05	−40	28	36
PDM	Val/Val > Val/Met	POV	Cuneus, L	17	686	3.73	−16	−92	4
	Val/Val > Met/Met	MENS	Sensorimotor, L	1	327	4.69	−18	−38	78
	Val/Met > Met/Met	MENS	Occipitotemporal, L	37	553	3.57	−54	−64	−10
	Met/Met > Val/Val	MENS	Pons, L	—	389	2.95	−10	−32	−20
		MENS	Hippocampus, L	—	—	3.04	−22	−22	−10
	Met/Met > Val/Met	POV	Pons, M	—	1720	3.92	0	−28	−28
		POV	Cuneus, L	19	471	3.55	−26	−92	26
		POV	Hippocampus, L	—	403	3.53	−28	−34	2
		POV	Putamen, L	—	—	3.29	−32	−22	0
		POV	Thalamus, L	—	—	3.14	−22	−28	2

Peak coordinates refer to the Montreal Neurological Institute (MNI) space. Significance was thresholded at the uncorrected voxel level *p* = 0.005, followed by the FDR-corrected cluster level *p* = 0.05.

BA, Brodmann area; CON, control; dlPFC, dorsolateral prefrontal cortex; L, left; M, medial; MENS, menstrual phase; Met, methionine; PCC, posterior cingulate cortex; PDM, primary dysmenorrhea; PFC, prefrontal cortex; POV, periovulatory phase; R, right; SMA, supplementary motor area; Val, valine.

**Table 4:** Peak MNI coordinates of the regions exhibiting significant resting-state functional connectivity with the PAG in PDM subjects compared with the healthy controls for each genotype.

Contrast of group	Genotype	Phase	Region, Laterality	BA	Cluster	*t* Score	Peak coordinate		
							*x*	*y*	*z*
PDM > CON	Val/Val	POV	Insula, L	13	338	4.46	−26	22	−8
		POV	Cerebellum, L	—	618	3.86	−10	−68	−48
	Val/Val	MENS	Sensorimotor, L	1	436	4.62	−18	−38	78
	Val/Met	POV	Premotor cortex, R	6	619	5.60	20	−6	64
	Val/Met	MENS	Occipitotemporal, L	37	556	3.73	−52	−62	−4
		MENS	Occipitotemporal, R	37	338	3.37	52	−68	−10
	Met/Met	POV	Pons, M	—	515	3.42	−6	−28	−28
CON > PDM	Val/Met	MENS	PFC, M	10	1136	3.28	10	60	6
	Val/Met	POV	PFC, M	10	2533	5.01	−4	52	4
		POV	Angular gyrus, L	39	535	4.28	−38	−74	30
		POV	PCC/precuneus, M	31	620	3.29	0	−70	28

Peak coordinates refer to the Montreal Neurological Institute (MNI) space. Significance was thresholded at the uncorrected voxel level *p* = 0.005, followed by the FDR-corrected cluster level *p* = 0.05.

BA, Brodmann area; CON, control; L, left; M, medial; MENS, menstrual phase; Met, methionine; PCC, posterior cingulate cortex; PDM, primary dysmenorrhea; PFC, prefrontal cortex; POV, periovulatory phase; R, right; Val, valine.

**Table 5:** *State*-related (MENS phase) and *trait*-related (POV phase) PAG functional connectivity covaries respectively with present* and recalled pain rating index of the PDM subjects.

Genotype	Phase	Region, Laterality	BA	Cluster	*t* Score	Peak coordinate		
						*x*	*y*	*z*
Val/Val	MENS	**Positive**						
		Precuneus, M	7	375	3.71	6	−48	48
	MENS	**Negative**						
		PFC, M	9	2284	4.91	4	58	32
		PFC, M	10	272	3.10	0	64	−10
		Secondary somatosensory, L	40	269	4.36	−54	−28	22
		Middle temporal gyrus, R	21	314	4.22	64	−20	−14
		Sensorimotor, L	4	258	3.69	−12	−30	80
		dlPFC, R	8	597	3.52	32	30	44
	POV	**Positive**						
		Precuneus, M	7	1773	5.72	−8	−68	30
		Angular gyrus, R	39	496	4.52	44	−54	48
		PFC, M	10	304	3.58	2	46	−2
	POV	**Negative**						
		vlPFC, R	46	299	5.30	46	40	12
		Orbitofrontal cortex, R	11	424	4.20	44	34	−12
Val/Met	POV	**Negative**						
		Sensorimotor, L	4	345	4.33	−52	−8	36
		Sensorimotor, R	4	532	4.24	46	−4	34
Met/Met	NA	NA	NA	NA	NA	NA	NA	NA

^*^Three PDM subjects did not complete the McGill Pain Questionnaire and were excluded from this correlation.

Peak coordinates refer to the Montreal Neurological Institute (MNI) space. Significance was thresholded at the uncorrected voxel level *p* = 0.005, followed by the FDR-corrected cluster level *p* = 0.05. No correlation found in the Met/Met genotype group. BA, Brodmann area; dlPFC, dorsolateral prefrontal cortex; L, left; M, medial; MENS, menstrual phase; Met, methionine; PDM, primary dysmenorrhea; PFC, prefrontal cortex; POV, periovulatory phase; R, right; Val, valine; vlPFC, ventrolateral prefrontal cortex; NA, not available.
