# Supplementary material for: The BDNF Val66Met polymorphism is associated with the functional connectivity dynamics of pain modulatory systems in primary dysmenorrhea
Source: Sci Rep. 2016 Mar 24;6:23639. doi: 10.1038/srep23639 (PMC4806293; doi:10.1038/srep23639)

The *BDNF* Val66Met polymorphism is associated with the functional connectivity dynamics of pain modulatory systems in primary dysmenorrhea

Shyh-Yuh Wei[1a,2a], Hsiang-Tai Chao[1b,2b], Cheng-Hao Tu[1a,2a], Ming-Wei Lin[1c], Wei-Chi Li[1a], Intan Low[1d], Horng-Der Shen[2c], Li-Fen Chen[1a,1d,2a**], and Jen-Chuen Hsieh[1a,2a*]

[1a]Institute of Brain Science, [1b]Department of Obstetrics and Gynecology, [1c]Institute of Public Health, [1d]Institute of Biomedical Informatics, School of Medicine, National Yang-Ming University, Taipei, Taiwan

[2a]Integrated Brain Research Unit, Division of Clinical Research, Department of Medical Research, [2b]Department of Obstetrics and Gynecology, [2c]Laboratory of Microbiology, Division of Basic Research, Department of Medical Research, Taipei Veterans General Hospital, Taipei, Taiwan

Supplementary Table S1. Results of repeated-measures ANOVA of Pain Catastrophizing Scale and Beck Depression Inventory: effects of group, *BDNF* genotype and menstrual cycle phase

	PDM			CON*			*p* value						
	Met/Met (n = 17)	Val/Met (n = 20)	Val/Val (n = 19)	Met/Met (n = 12)	Val/Met (n = 29)	Val/Val (n = 19)	Main effect of phase	Main effect of group	Main effect of genotype	Interaction between phase and group	Interaction between phase and genotype	Interaction between group and genotype	Interaction among phase, group and genotype
Pain Catastrophizing Scale													
MENS	22.7 (10.31)	18.9 (12.40)	23.6 (10.17)	5.2 (5.91)	5.9 (7.18)	3.3 (3.87)	0.083	0.000	0.861	0.008	0.794	0.076	0.466
POV	17.8 (8.97)	16.4 (11.53)	21.7 (11.24)	6.3 (7.27)	7.1 (7.89)	2.9 (6.30)							
Beck Depression Inventory													
MENS	11.9 (7.12)	11.7 (10.84)	11.7 (6.07)	4.5 (2.88)	4.1 (5.37)	3.6 (4.25)	0.000	0.000	0.686	0.000	0.536	0.706	0.307
POV	6.2 (6.24)	7.3 (9.09)	6.1 (6.42)	7.3 (6.03)	4.1 (6.29)	3.3 (3.58)							

* Two control subjects who did not complete the Pain Catastrophizing Scale (2 Val/Val) and Beck Depression Inventory (1 Val/Val, 1 Val/Met) who were excluded from this calculation.

Abbreviations: *BDNF*, brain-derived neurotrophic factor; PDM, primary dysmenorrhea; CON, control; MENS, menstrual phase; POV, periovulatory phase; Val, valine; Met, methionine. The data are presented as the means (SD).

Supplementary Table S2. Results of repeated-measures ANOVA of gonadal hormone levels: effects of group, *BDNF* genotype and menstrual cycle phase

	PDM			CON			*p* value						
	Met/Met (*n* = 17)	Val/Met (*n* = 20)	Val/Val (*n* = 19)	Met/Met (*n* = 12)	Val/Met (*n* = 29)	Val/Val (*n* = 19)	Main effect of phase	Main effect of group	Main effect of genotype	Interaction between phase and group	Interaction between phase and genotype	Interaction between group and genotype	Interaction among phase, group and genotype
Estradiol (pg/mL)													
MENS	32.1 (15.12)	36.4 (18.39)	29.1 (16.66)	38.8 (21.24)	34.0 (17.45)	35.4 (16.61)	0.000	0.728	0.061	0.476	0.070	0.745	0.902
POV	149.4 (105.84)	189.6 (115.24)	117.8 (78.95)	137.1 (106.90)	164.1 (131.95)	121.8 (102.33)							
Progesterone (ng/mL)													
MENS	0.4 (0.30)	0.5 (0.30)	0.5 (0.48)	1.4 (3.47)	0.4 (0.26)	0.5 (0.32)	0.087	0.089	0.712	0.482	0.197	0.476	0.282
POV	0.5 (0.45)	0.8 (0.67)	1.0 (1.62)	0.9 (2.31)	2.5 (5.32)	1.1 (1.55)							
Testosterone (ng/mL)													
MENS	0.5 (0.26)	0.4 (0.25)	0.3 (0.13)	0.4 (0.19)	0.4 (0.26)	0.3 (0.16)	0.000	0.588	0.043	0.436	0.024	0.381	0.286
POV	0.6 (0.38)	0.5 (0.29)	0.4 (0.19)	0.5 (0.31)	0.4 (0.16)	0.5 (0.16)							

Abbreviations: *BDNF*, brain-derived neurotrophic factor; PDM, primary dysmenorrhea; CON, control; MENS, menstrual phase; POV, periovulatory phase; Val, valine; Met, methionine. The data are presented as the means (SD).

Supplementary Table S3. Regions exhibit significant (FWE-corrected voxel level *p*=0.05 and FWE-corrected cluster level *p*=0.05) resting-state functional connectivity with the PAG for different genotypes in each group during menstruation (MENS) and periovulatory (POV) phases

Group	Phase	Laterality	Region	Brodmann area	Cluster size	*t* Score	Peak coordinate (x, y, z)
Val/Val controls	POV	Left Brainstem	Red Nucleus		14098	50.75	-4 -26 -14
		Right Cerebrum	Precuneus	31	761	7.24	16 -50 34
		Left Cerebrum	Middle Frontal Gyrus	10	65	6.60	-30 62 14
		Left Cerebrum	Precuneus	7	67	6.43	-16 -50 46
		Left Cerebrum	Precuneus	7	43	6.26	-10 -70 66
		Right Cerebrum	Middle Temporal Gyrus	21	52	6.24	46 -2 -24
		Left Cerebrum	Superior Temporal Gyrus	39	118	6.23	-48 -56 22
		Right Cerebellum	Tuber		24	6.19	56 -58 -36
		Left Cerebrum	Inferior Temporal Gyrus	20	29	6.07	-54 -46 -14
		Right Cerebrum	Fusiform Gyrus	20	37	5.93	54 -26 -32
		Left Cerebrum	Superior Temporal Gyrus	41	18	5.92	-44 -34 4
		Right Cerebellum	Pyramis		29	5.91	12 -70 -34
		Left Cerebellum	Inferior Semi-Lunar Lobule		12	5.89	-50 -66 -50
		Right Cerebrum	Medial Frontal Gyrus	6	30	5.88	16 6 56
		Left Cerebrum	Uncus	20	37	5.84	-32 -22 -44
		Left Cerebrum	Precentral Gyrus	6	15	5.83	-34 -4 40
		Right Cerebrum	Precuneus	7	33	5.79	16 -64 58
		Right Cerebrum	Supramarginal Gyrus	40	25	5.74	42 -40 32
		Right Cerebrum	Cuneus	18	12	5.69	14 -104 0
		Right Cerebrum	Inferior Temporal Gyrus	20	6	5.66	42 -22 -38
		Left Cerebrum	Precuneus	7	27	5.61	-22 -74 52
		Left Cerebellum	Culmen		5	5.57	-38 -40 -38
		Left Cerebrum	Middle Temporal Gyrus	39	26	5.56	-50 -76 26
		Right Cerebrum	Rectal Gyrus	11	14	5.55	10 18 -26
		Left Cerebrum	Parahippocampal Gyrus	30	8	5.54	-26 -52 6
		Left Cerebrum	Precuneus	7	68	5.53	-2 -56 54
		Left Cerebrum	Superior Temporal Gyrus	41	5	5.46	-44 -44 4
		Right Cerebrum	Inferior Temporal Gyrus	20	6	5.45	48 -12 -48
		Left Cerebellum	Uvula		11	5.44	-6 -66 -38
		Left Cerebrum	Cingulate Gyrus	32	10	5.44	-22 6 48

Group	Phase	Laterality	Region	Brodmann area	Cluster size	*t* Score	Peak coordinate (x, y, z)
		Right Cerebrum	Inferior Temporal Gyrus	20	3	5.43	36 -4 -48
		Right Cerebellum	Pyramis		1	5.43	50 -74 -44
		Right Cerebrum	Precuneus	7	5	5.42	24 -56 42
		Right Cerebrum	Cingulate Gyrus	24	3	5.42	16 -8 44
		Left Cerebrum	Middle Temporal Gyrus	39	8	5.41	-42 -74 16
		Right Cerebrum	Angular Gyrus	39	17	5.39	46 -70 30
		Left Cerebrum	Inferior Parietal Lobule	40	1	5.39	-46 -48 30
		Right Cerebrum	Inferior Temporal Gyrus	20	1	5.38	52 -6 -46
		Right Cerebrum	Cingulate Gyrus	32	1	5.36	16 14 30
		Right Cerebrum	Inferior Temporal Gyrus	20	1	5.35	58 -10 -36
		Right Cerebrum	Precuneus	39	2	5.35	40 -74 32
		Left Cerebrum	Precentral Gyrus	6	9	5.35	-34 -14 38
		Right Cerebrum	Precuneus	7	2	5.35	12 -60 48
		Right Cerebellum	Pyramis		16	5.34	28 -66 -40
		Left Cerebrum	Lentiform Nucleus (Putamen)		4	5.31	-26 0 10
		Right Cerebrum	Paracentral Lobule	5	2	5.31	10 -40 60
		Right Cerebrum	Medial Frontal Gyrus	32	1	5.31	20 8 48
		Right Cerebellum	Declive		4	5.28	12 -72 -22
		Right Cerebrum	Cingulate Gyrus	32	1	5.28	24 14 36
		Right Cerebellum	Culmen		3	5.27	36 -38 -38
		Right Cerebrum	Paracentral Lobule	6	1	5.27	6 -34 56
		Right Cerebrum	Superior Frontal Gyrus	6	2	5.26	12 24 68
		Left Cerebrum	Superior Temporal Gyrus	22	1	5.25	-38 -52 16
		Right Cerebrum	Inferior Temporal Gyrus	20	1	5.25	56 -16 -34
		Right Cerebrum	Rectal Gyrus	11	3	5.25	2 16 -28
		Right Cerebrum	Cuneus	17	1	5.24	12 -100 2
		Left Cerebrum	Parahippocampal Gyrus	19	1	5.24	-32 -44 -4
		Left Cerebellum	Cerebellar Tonsil		1	5.22	-54 -60 -42
		Right Cerebrum	Middle Frontal Gyrus	6	2	5.22	30 8 68
		Right Cerebellum	Inferior Semi-Lunar Lobule		3	5.21	30 -70 -48
		Right Cerebrum	Medial Frontal Gyrus	32	1	5.19	24 10 44
		Left Cerebrum	Lentiform Nucleus (Putamen)		1	5.19	-30 -2 4

Group	Phase	Laterality	Region	Brodmann area	Cluster size	*t* Score	Peak coordinate (x, y, z)
		Left Cerebellum	Cerebellar Tonsil		1	5.19	-48 -58 -48
		Left Cerebrum	Sub-Gyral	20	1	5.18	-40 -14 -22
		Right Cerebrum	Inferior Parietal Lobule	40	2	5.18	50 -36 26
		Left Cerebrum	Superior Temporal Gyrus	22	1	5.18	-50 -26 2
		Right Cerebrum	Uncus	28	2	5.17	14 -2 -32
		Left Cerebrum	Inferior Frontal Gyrus	46	1	5.17	-54 34 14
		Left Cerebrum	Lentiform Nucleus (Putamen)		1	5.17	-28 -12 12
		Right Cerebrum	Inferior Temporal Gyrus	20	1	5.17	58 -58 -20
		Right Cerebellum	Pyramis		1	5.16	22 -60 -36
		Left Cerebrum	Amygdala		1	5.16	-32 -6 -20
		Left Cerebrum	Medial Frontal Gyrus	32	1	5.16	-18 12 46
		Right Cerebrum	Cingulate Gyrus	31	1	5.15	26 -44 32
		Left Cerebrum	Middle Frontal Gyrus	6	1	5.15	-20 -22 62
		Right Cerebrum	Lentiform Nucleus (Putamen)		1	5.14	32 -4 10
		Right Cerebrum	Superior Temporal Gyrus	38	1	5.14	36 16 -32
		Left Cerebrum	Superior Parietal Lobule	7	1	5.14	-26 -68 56
		Left Cerebrum	Precuneus	7	1	5.14	-18 -64 56
		Right Cerebrum	Precuneus	39	1	5.13	42 -72 30
		Right Cerebellum	Dentate		1	5.12	20 -62 -34
		Right Cerebrum	Lateral Globus Pallidus		1	5.12	20 -6 -4
		Right Cerebrum	Middle Temporal Gyrus	39	1	5.11	44 -68 28
		Right Cerebrum	Superior Parietal Lobule	7	1	5.11	30 -60 60
Val/Val controls	MENS	Left Brainstem	Red Nucleus		14200	60.45	-4 -26 -14
		Right Cerebrum	Superior Frontal Gyrus	6	2932	7.77	12 10 64
		Right Cerebrum	Superior Frontal Gyrus	8	456	6.88	24 40 54
		Left Cerebrum	Insula	13	111	6.81	-34 -44 20
		Right Cerebrum	Middle Temporal Gyrus	21	67	6.36	66 -34 -18
		Right Cerebrum	Cuneus	18	33	6.29	14 -106 0
		Right Cerebrum	Inferior Frontal Gyrus	47	64	6.20	28 26 -16
		Left Cerebrum	Superior Frontal Gyrus	10	65	6.19	-32 54 24
		Left Cerebrum	Superior Frontal Gyrus	6	52	6.16	-20 16 68

Group	Phase	Laterality	Region	Brodmann area	Cluster size	*t* Score	Peak coordinate (x, y, z)
		Left Cerebrum	Inferior Frontal Gyrus	13	98	6.14	-36 10 -12
		Left Cerebellum	Declive		24	6.04	-54 -58 -28
		Right Cerebrum	Insula	13	24	5.99	30 -14 28
		Right Cerebrum	Medial Frontal Gyrus	10	42	5.87	18 70 10
		Left Cerebrum	Superior Temporal Gyrus	13	7	5.77	-46 -48 24
		Right Cerebrum	Uncus	28	24	5.74	28 6 -24
		Right Cerebrum	Superior Temporal Gyrus	41	26	5.71	34 -36 14
		Right Cerebrum	Superior Temporal Gyrus	38	53	5.69	46 10 -10
		Right Cerebellum	Cerebellar Tonsil		25	5.69	46 -48 -42
		Right Cerebellum	Inferior Semi-Lunar Lobule		10	5.66	48 -72 -50
		Left Cerebrum	Inferior Temporal Gyrus	20	5	5.64	-52 -24 -36
		Left Cerebrum	Superior Frontal Gyrus	8	35	5.61	-28 28 58
		Left Cerebrum	Cuneus	17	6	5.58	-10 -106 -6
		Right Cerebrum	Insula	13	8	5.50	28 -36 18
		Left Cerebrum	Inferior Temporal Gyrus	20	4	5.50	-68 -24 -22
		Right Cerebrum	Superior Temporal Gyrus	13	4	5.49	42 -50 26
		Left Cerebrum	Parahippocampal Gyrus	30	17	5.46	-30 -56 6
		Right Cerebrum	Superior Temporal Gyrus	39	5	5.45	34 -56 30
		Right Cerebrum	Subcallosal Gyrus	25	5	5.45	8 10 -16
		Right Cerebellum	Declive		5	5.44	20 -62 -20
		Left Cerebrum	Cingulate Gyrus	24	10	5.43	-6 4 38
		Left Cerebrum	Superior Frontal Gyrus	6	1	5.38	-26 -6 74
		Left Cerebrum	Superior Frontal Gyrus	6	3	5.35	-10 -4 78
		Left Cerebrum	Postcentral Gyrus	3	1	5.33	-30 -28 42
		Right Cerebrum	Inferior Temporal Gyrus	20	3	5.33	52 -30 -20
		Right Cerebrum	Middle Temporal Gyrus	21	1	5.32	66 -4 -14
		Right Cerebrum	Cuneus	17	2	5.32	4 -100 -6
		Right Cerebrum	Superior Temporal Gyrus	13	1	5.30	44 -48 24
		Right Cerebrum	Superior Frontal Gyrus	10	2	5.30	12 58 -10
		Right Cerebrum	Superior Frontal Gyrus	9	7	5.30	12 62 36
		Right Cerebrum	Posterior Cingulate	29	1	5.27	4 -52 6
		Right Cerebellum	Pyramis		4	5.26	30 -58 -36

Group	Phase	Laterality	Region	Brodmann area	Cluster size	*t* Score	Peak coordinate (x, y, z)
		Right Cerebrum	Middle Temporal Gyrus	21	1	5.26	62 2 -28
		Left Cerebrum	Parahippocampal Gyrus	30	1	5.25	-20 -40 4
		Right Cerebrum	Uncus	20	1	5.24	28 -20 -42
		Right Cerebellum	Tuber		1	5.23	56 -60 -32
		Left Cerebrum	Cuneus	18	1	5.22	-16 -106 -8
		Left Cerebrum	Superior Frontal Gyrus	6	2	5.22	-28 -4 72
		Left Cerebrum	Cuneus	18	3	5.21	-22 -104 -6
		Right Cerebellum	Cerebellar Tonsil		3	5.21	24 -54 -42
		Right Cerebrum	Subcallosal Gyrus	25	1	5.19	8 16 -16
		Left Cerebrum	Superior Frontal Gyrus	6	1	5.18	-22 8 72
		Left Cerebrum	Middle Frontal Gyrus	9	1	5.18	-52 30 34
		Left Cerebrum	Insula	13	1	5.18	-34 16 16
		Right Cerebrum	Inferior Frontal Gyrus	11	2	5.18	24 38 -22
		Left Cerebrum	Superior Frontal Gyrus	6	1	5.17	-22 -6 76
		Left Cerebrum	Medial Frontal Gyrus	10	1	5.15	-6 52 -8
		Left Cerebellum	Pyramis		1	5.15	-20 -70 -40
		Left Cerebellum	Tuber		1	5.14	-28 -90 -40
		Right Cerebrum	Superior Frontal Gyrus	10	1	5.14	20 66 18
		Right Cerebrum	Lingual Gyrus	17	1	5.14	4 -96 -8
		Left Cerebellum	Tuber		2	5.14	-44 -66 -38
		Left Cerebellum	Tuber		1	5.14	-40 -64 -36
Val/Met controls	POV	Left Brainstem	Red Nucleus		46348	59.31	-4 -26 -14
		Right Cerebrum	Inferior Parietal Lobule	40	1104	7.76	42 -60 38
		Left Cerebrum	Middle Temporal Gyrus	21	296	6.27	-66 -18 -14
		Left Cerebrum	Inferior Temporal Gyrus	20	12	6.26	-38 -2 -50
		Left Cerebrum	Rectal Gyrus	11	102	6.19	-12 18 -24
		Right Cerebrum	Middle Temporal Gyrus	21	57	5.89	70 -24 -6
		Right Cerebellum	Cerebellar Tonsil		21	5.87	38 -52 -46
		Right Cerebrum	Cingulate Gyrus	24	14	5.84	26 -16 36
		Right Cerebrum	Insula	13	20	5.64	44 -2 -8
		Left Cerebrum	Inferior Frontal Gyrus	47	11	5.63	-38 34 -20

Group	Phase	Laterality	Region	Brodmann area	Cluster size	*t* Score	Peak coordinate (x, y, z)
		Left Cerebrum	Middle Temporal Gyrus	21	42	5.55	-58 -6 -26
		Left Cerebellum	Tuber		16	5.53	-52 -54 -34
		Right Cerebrum	Parahippocampal Gyrus	36	2	5.49	36 -24 -32
		Left Cerebrum	Inferior Temporal Gyrus	20	5	5.47	-52 -10 -46
		Left Cerebrum	Middle Temporal Gyrus	21	16	5.39	-48 2 -28
		Left Cerebellum	Inferior Semi-Lunar Lobule		1	5.39	-12 -70 -58
		Left Cerebrum	Fusiform Gyrus	20	1	5.35	-50 -30 -28
		Right Cerebrum	Middle Frontal Gyrus	46	1	5.26	46 38 22
		Left Cerebrum	Superior Temporal Gyrus	21	1	5.25	-50 -24 -4
		Right Cerebrum	Parahippocampal Gyrus	35	3	5.24	24 -12 -26
		Right Cerebrum	Middle Temporal Gyrus	21	2	5.23	58 -28 -18
		Right Cerebellum	Pyramis		1	5.22	6 -90 -38
		Left Cerebrum	Uncus	36	1	5.22	-20 -6 -38
		Right Cerebellum	Inferior Semi-Lunar Lobule		1	5.21	38 -74 -56
		Right Cerebrum	Uncus	38	1	5.18	20 4 -40
		Left Cerebrum	Inferior Temporal Gyrus	20	2	5.18	-56 -12 -42
		Right Cerebrum	Inferior Frontal Gyrus	47	3	5.18	44 24 -4
		Left Cerebrum	Uncus	36	1	5.17	-16 -6 -40
		Left Cerebrum	Superior Frontal Gyrus	6	1	5.16	-10 24 68
		Left Cerebrum	Insula	13	1	5.15	-30 -6 26
		Left Cerebrum	Middle Frontal Gyrus	10	1	5.13	-32 58 -8
		Left Cerebrum	Fusiform Gyrus	20	1	5.13	-44 -4 -24
		Left Cerebrum	Middle Frontal Gyrus	10	1	5.12	-32 48 24
		Right Cerebellum	Cerebellar Tonsil		1	5.11	32 -50 -46
		Left Cerebellum	Tuber		1	5.11	-50 -58 -32
Val/Met controls	MENS	Left Brainstem	Red Nucleus		52715	72.13	-4 -26 -14
		Right Cerebrum	Inferior Frontal Gyrus	46	149	7.03	34 32 14
		Right Cerebrum	Supramarginal Gyrus	40	690	6.64	64 -50 34
		Left Cerebrum	Inferior Frontal Gyrus	47	142	6.57	-30 32 -12
		Left Cerebrum	Superior Occipital Gyrus	19	92	6.36	-40 -84 36
		Left Cerebrum	Insula	13	57	5.97	-34 26 12

Group	Phase	Laterality	Region	Brodmann area	Cluster size	*t* Score	Peak coordinate (x, y, z)
		Right Cerebrum	Caudate		18	5.74	36 -42 4
		Right Cerebrum	Insula	13	9	5.68	32 16 14
		Right Cerebrum	Inferior Parietal Lobule	39	34	5.68	54 -66 42
		Right Cerebrum	Cuneus	18	24	5.67	10 -104 4
		Right Cerebrum	Fusiform Gyrus	18	14	5.62	20 -98 -20
		Left Cerebrum	Middle Frontal Gyrus	6	4	5.54	-34 10 66
		Right Cerebrum	Caudate		15	5.51	28 -42 12
		Right Cerebrum	Inferior Frontal Gyrus	45	4	5.47	58 16 2
		Right Cerebrum	Superior Parietal Lobule	7	11	5.42	44 -68 52
		Right Cerebrum	Cingulate Gyrus	31	3	5.39	22 -32 42
		Left Cerebrum	Superior Frontal Gyrus	6	2	5.38	-30 2 70
		Right Cerebrum	Middle Frontal Gyrus	6	2	5.36	18 -8 58
		Right Cerebrum	Cingulate Gyrus	31	5	5.34	18 -40 40
		Left Cerebrum	Middle Frontal Gyrus	8	4	5.34	-36 18 40
		Right Cerebrum	Anterior Cingulate	25	1	5.28	6 10 -10
		Right Cerebrum	Inferior Frontal Gyrus	11	2	5.26	16 30 -20
		Right Cerebrum	Middle Frontal Gyrus	10	3	5.26	32 40 18
		Left Cerebrum	Inferior Parietal Lobule	40	2	5.24	-62 -42 44
		Left Cerebrum	Middle Frontal Gyrus	11	4	5.24	-32 42 -2
		Left Cerebrum	Middle Frontal Gyrus	10	1	5.23	-28 36 14
		Left Cerebrum	Middle Frontal Gyrus	8	1	5.22	-48 26 44
		Left Cerebrum	Insula	13	1	5.21	-40 6 12
		Right Cerebrum	Superior Frontal Gyrus	9	1	5.21	14 62 36
		Left Cerebrum	Sub-Gyral		1	5.21	-30 44 2
		Right Cerebrum	Middle Temporal Gyrus	21	1	5.19	60 -22 -12
		Left Cerebrum	Superior Frontal Gyrus	10	1	5.18	-20 42 24
		Left Cerebrum	Middle Frontal Gyrus	6	1	5.18	-22 22 54
		Right Cerebrum	Superior Frontal Gyrus	9	1	5.18	12 64 34
		Right Cerebrum	Cuneus	19	1	5.17	18 -96 28
		Right Cerebrum	Inferior Parietal Lobule	40	1	5.17	48 -48 60
		Right Cerebrum	Cingulate Gyrus	31	1	5.15	24 -34 32
		Right Cerebrum	Inferior Frontal Gyrus	47	1	5.14	52 40 -14

Group	Phase	Laterality	Region	Brodmann area	Cluster size	*t* Score	Peak coordinate (x, y, z)
		Left Cerebrum	Middle Frontal Gyrus	46	1	5.13	-44 46 26
Met/Met controls	POV	Left Brainstem	Red Nucleus		1210	40.39	-4 -26 -14
		Left Cerebrum	Uncus	20	192	7.23	-24 0 -44
		Right Cerebrum	Middle Frontal Gyrus	6	81	7.13	36 -8 52
		Right Cerebrum	Middle Temporal Gyrus	21	66	6.73	66 -28 -12
		Right Cerebrum	Cingulate Gyrus	24	43	6.51	8 0 44
		Left Cerebrum	Precentral Gyrus	6	53	6.51	-54 2 18
		Right Cerebrum	Superior Temporal Gyrus	22	19	6.48	70 -40 12
		Right Cerebrum	Medial Frontal Gyrus	6	27	6.40	12 -2 58
		Right Cerebrum	Fusiform Gyrus	37	96	6.31	40 -56 -14
		Left Cerebrum	Cingulate Gyrus	32	30	6.20	-18 14 28
		Left Cerebrum	Parahippocampal Gyrus	19	55	5.93	-40 -40 -8
		Right Cerebrum	Inferior Occipital Gyrus	19	38	5.86	38 -76 -6
		Left Cerebrum	Middle Frontal Gyrus	6	8	5.81	-26 -8 48
		Right Cerebrum	Insula	13	11	5.77	30 -38 16
		Right Cerebrum	Medial Frontal Gyrus	6	9	5.75	2 -16 60
		Left Cerebellum	Culmen		44	5.73	-20 -48 -26
		Right Cerebrum	Middle Frontal Gyrus	47	9	5.69	38 40 -2
		Right Cerebrum	Middle Frontal Gyrus	9	9	5.53	58 14 34
		Left Cerebrum	Fusiform Gyrus	20	6	5.52	-54 -28 -34
		Left Cerebrum	Anterior Cingulate	32	5	5.47	-6 24 -10
		Right Cerebrum	Cingulate Gyrus	24	4	5.47	16 -14 36
		Right Cerebrum	Parahippocampal Gyrus	19	2	5.47	42 -42 -6
		Right Cerebrum	Uncus	28	4	5.45	16 10 -32
		Left Cerebrum	Sub-Gyral	6	4	5.45	-24 -4 62
		Left Cerebrum	Middle Occipital Gyrus	18	3	5.42	-36 -86 -6
		Left Cerebrum	Superior Temporal Gyrus	38	7	5.40	-28 10 -28
		Right Cerebrum	Cingulate Gyrus	24	1	5.38	12 -10 32
		Right Cerebrum	Inferior Temporal Gyrus	20	3	5.38	32 -12 -46
		Left Cerebrum	Fusiform Gyrus	20	4	5.38	-46 -22 -22
		Left Cerebrum	Fusiform Gyrus	37	7	5.36	-38 -60 -12

Group	Phase	Laterality	Region	Brodmann area	Cluster size	*t* Score	Peak coordinate (x, y, z)
		Right Cerebrum	Subcallosal Gyrus	25	2	5.32	4 12 -14
		Right Cerebrum	Uncus	36	3	5.29	22 -6 -34
		Right Brainstem	Substania Nigra		5	5.27	18 -20 -10
		Right Cerebrum	Superior Frontal Gyrus	9	1	5.19	14 52 26
		Left Cerebellum	Declive		2	5.18	-30 -54 -16
		Right Cerebellum	Culmen		1	5.17	6 -64 -14
		Left Cerebrum	Middle Occipital Gyrus	19	2	5.15	-48 -82 0
		Left Cerebrum	Fusiform Gyrus	37	1	5.13	-32 -50 -16
		Right Cerebrum	Claustrum		1	5.13	24 26 8
		Left Cerebrum	Inferior Parietal Lobule	40	2	5.13	-54 -50 48
		Left Cerebrum	Inferior Temporal Gyrus	20	3	5.12	-52 2 -44
		Left Cerebrum	Precuneus	19	1	5.12	-32 -82 44
		Left Cerebrum	Inferior Frontal Gyrus	47	1	5.11	-50 24 -2
Met/Met controls	MENS	Left Brainstem	Red Nucleus		5098	46.89	-4 -26 -14
		Right Cerebrum	Posterior Cingulate	29	632	7.58	16 -50 12
		Left Cerebrum	Cingulate Gyrus	32	329	6.83	-4 20 46
		Left Cerebrum	Cingulate Gyrus	24	96	6.67	-20 4 48
		Left Cerebrum	Middle Frontal Gyrus	9	180	6.30	-40 26 36
		Left Cerebellum	Declive		88	6.29	-34 -64 -28
		Left Cerebrum	Supramarginal Gyrus	40	163	6.29	-38 -48 36
		Left Cerebrum	Middle Occipital Gyrus	18	33	6.25	-36 -84 -14
		Left Cerebellum	Pyramis		63	6.07	-10 -86 -36
		Right Cerebrum	Anterior Cingulate	32	45	5.95	8 40 26
		Right Cerebrum	Caudate		67	5.93	20 -4 22
		Left Cerebrum	Middle Frontal Gyrus	6	20	5.89	-20 -12 48
		Right Cerebrum	Middle Frontal Gyrus	8	60	5.87	48 24 44
		Left Cerebrum	Rectal Gyrus	11	13	5.81	-10 16 -32
		Right Cerebrum	Medial Frontal Gyrus	10	7	5.79	22 46 -8
		Left Cerebrum	Fusiform Gyrus	20	2	5.73	-46 -32 -32
		Left Cerebrum	Insula	47	7	5.71	-32 16 -2
		Left Cerebrum	Precuneus	19	78	5.67	-34 -74 32

Group	Phase	Laterality	Region	Brodmann area	Cluster size	*t* Score	Peak coordinate (x, y, z)
		Right Cerebellum	Tuber		79	5.60	44 -62 -32
		Right Cerebrum	Middle Frontal Gyrus	46	17	5.58	38 30 26
		Left Cerebrum	Cingulate Gyrus	31	4	5.57	-22 -50 24
		Right Cerebrum	Inferior Frontal Gyrus	47	17	5.55	34 20 -6
		Left Cerebrum	Superior Frontal Gyrus	8	28	5.55	-8 14 60
		Left Cerebrum	Parahippocampal Gyrus	30	15	5.45	-10 -46 2
		Left Cerebrum	Precuneus	7	17	5.39	-8 -68 54
		Right Cerebrum	Medial Frontal Gyrus	6	7	5.37	20 2 52
		Right Cerebrum	Middle Frontal Gyrus	46	2	5.36	40 30 20
		Left Cerebrum	Cingulate Gyrus	31	2	5.33	-18 -58 28
		Right Cerebrum	Precuneus	7	6	5.31	12 -56 48
		Left Cerebrum	Inferior Frontal Gyrus	46	6	5.29	-46 28 16
		Right Cerebrum	Cuneus	17	4	5.28	16 -98 0
		Right Cerebrum	Cingulate Gyrus	24	4	5.27	6 2 28
		Left Cerebrum	Cingulate Gyrus	31	3	5.26	-20 -54 24
		Left Cerebrum	Thalamus		1	5.23	-24 -26 0
		Left Cerebrum	Middle Frontal Gyrus	6	1	5.22	-34 6 48
		Right Cerebrum	Superior Frontal Gyrus	10	3	5.22	32 64 -6
		Right Cerebrum	Middle Frontal Gyrus	6	1	5.22	26 6 50
		Right Cerebrum	Precentral Gyrus	9	1	5.21	34 8 40
		Left Cerebrum	Caudate		1	5.18	-34 -38 0
		Right Cerebrum	Anterior Cingulate	24	1	5.18	12 36 0
		Right Cerebrum	Hypothalamus		1	5.17	10 -2 -6
		Right Cerebellum	Culmen of Vermis		1	5.17	2 -66 -4
		Right Cerebrum	Cingulate Gyrus	24	1	5.17	8 6 28
		Right Cerebrum	Lingual Gyrus	18	2	5.17	4 -94 -20
		Right Cerebellum	Inferior Semi-Lunar Lobule		1	5.16	34 -66 -48
		Right Cerebellum	Cerebellar Tonsil		1	5.15	38 -66 -44
		Left Cerebrum	Putamen		1	5.14	-16 4 12
		Right Cerebrum	Cingulate Gyrus	24	1	5.14	10 0 30
Val/Val PDM	POV	Left Brainstem	Red Nucleus		15395	46.21	-4 -26 -14

Group	Phase	Laterality	Region	Brodmann area	Cluster size	*t* Score	Peak coordinate (x, y, z)
		Right Cerebrum	Cingulate Gyrus	31	42	6.61	26 -28 40
		Right Cerebrum	Inferior Frontal Gyrus	47	114	6.48	38 34 -2
		Right Cerebrum	Anterior Cingulate	32	36	6.41	16 34 24
		Right Cerebrum	Insula	13	88	6.38	34 -12 22
		Left Cerebrum	Superior Frontal Gyrus	10	96	6.27	-18 68 24
		Left Cerebrum	Middle Frontal Gyrus	6	29	6.21	-48 12 52
		Left Cerebrum	Middle Frontal Gyrus	9	55	6.09	-46 30 38
		Right Cerebrum	Anterior Cingulate	32	85	6.02	10 42 14
		Right Cerebrum	Superior Frontal Gyrus	6	6	6.01	10 20 70
		Left Cerebrum	Anterior Cingulate	32	186	5.89	-10 34 20
		Right Cerebrum	Insula	13	48	5.88	34 -42 16
		Left Cerebrum	Cingulate Gyrus	31	20	5.86	-24 -28 40
		Left Cerebrum	Uncus	36	87	5.80	-26 2 -36
		Left Cerebrum	Cingulate Gyrus	31	65	5.79	-10 -42 40
		Left Cerebrum	Middle Frontal Gyrus	11	13	5.76	-34 46 -8
		Right Cerebrum	Posterior Cingulate	23	29	5.71	2 -32 24
		Left Cerebrum	Superior Parietal Lobule	7	57	5.67	-42 -64 50
		Left Cerebrum	Uncus	28	26	5.66	-26 -12 -32
		Left Cerebrum	Middle Frontal Gyrus	6	15	5.66	-30 14 66
		Right Cerebrum	Claustrum		36	5.61	28 18 -8
		Left Cerebellum	Cerebellar Tonsil		40	5.60	-32 -58 -58
		Right Cerebrum	Superior Frontal Gyrus	10	8	5.56	20 66 28
		Right Cerebrum	Claustrum		6	5.52	32 0 18
		Left Cerebrum	Insula	13	4	5.52	-32 -8 26
		Left Cerebellum	Inferior Semi-Lunar Lobule		8	5.49	-40 -74 -54
		Right Cerebrum	Inferior Frontal Gyrus	47	1	5.47	18 12 -16
		Left Cerebrum	Inferior Parietal Lobule	40	3	5.44	-52 -44 40
		Left Cerebrum	Inferior Temporal Gyrus	20	5	5.44	-42 -4 -46
		Left Cerebrum	Putamen		13	5.44	-24 -8 10
		Right Cerebrum	Superior Frontal Gyrus	6	4	5.43	26 16 68
		Left Cerebrum	Cingulate Gyrus	31	2	5.43	-20 -44 38
		Right Cerebellum	Inferior Semi-Lunar Lobule		10	5.40	28 -74 -48

Group	Phase	Laterality	Region	Brodmann area	Cluster size	*t* Score	Peak coordinate (x, y, z)
		Right Cerebrum	Precuneus	7	10	5.39	14 -64 32
		Right Cerebrum	Caudate		1	5.38	28 -34 12
		Left Cerebrum	Anterior Cingulate	24	6	5.34	-6 40 2
		Right Cerebrum	Cingulate Gyrus	31	2	5.32	22 -46 30
		Left Cerebrum	Cingulate Gyrus	31	2	5.32	-24 -30 44
		Right Cerebrum	Insula	13	1	5.31	42 2 18
		Left Cerebrum	Superior Frontal Gyrus	8	4	5.30	-2 40 62
		Right Cerebrum	Precentral Gyrus	4	1	5.27	26 -24 50
		Right Cerebrum	Cingulate Gyrus	32	1	5.25	22 14 30
		Right Cerebrum	Anterior Cingulate		2	5.25	8 42 2
		Right Cerebrum	Inferior Frontal Gyrus	47	4	5.23	22 24 -20
		Left Cerebrum	Inferior Parietal Lobule	40	3	5.21	-48 -54 50
		Left Cerebrum	Cingulate Gyrus	31	8	5.20	-14 -58 26
		Right Cerebrum	Cingulate Gyrus	31	2	5.20	6 -44 34
		Right Cerebellum	Pyramis		2	5.19	24 -70 -42
		Left Cerebrum	Parahippocampal Gyrus	35	1	5.18	-26 -22 -28
		Right Cerebrum	Cingulate Gyrus	31	2	5.18	22 -48 34
		Right Cerebrum	Superior Frontal Gyrus	8	3	5.18	36 16 58
		Left Cerebrum	Anterior Cingulate	32	1	5.16	-10 44 6
		Left Cerebrum	Cingulate Gyrus	31	2	5.16	-4 -30 40
		Right Cerebrum	Cingulate Gyrus	32	1	5.16	20 12 32
		Left Cerebrum	Cingulate Gyrus	24	2	5.16	-22 -22 44
		Right Cerebrum	Precentral Gyrus	9	1	5.16	34 10 38
		Left Cerebrum	Inferior Parietal Lobule	40	1	5.15	-46 -54 44
		Right Cerebrum	Superior Frontal Gyrus	10	1	5.15	14 72 16
		Left Cerebellum	Inferior Semi-Lunar Lobule		1	5.14	-38 -64 -48
		Left Cerebrum	Superior Frontal Gyrus	6	1	5.14	-8 34 64
		Left Cerebrum	Middle Temporal Gyrus	21	1	5.14	-48 10 -38
		Right Cerebrum	Putamen		1	5.14	16 10 6
		Right Cerebrum	Inferior Temporal Gyrus	20	1	5.14	62 -28 -30
		Left Cerebrum	Parahippocampal Gyrus		1	5.13	-12 -48 -2
		Left Cerebrum	Cingulate Gyrus	9	1	5.13	-12 26 36

Group	Phase	Laterality	Region	Brodmann area	Cluster size	*t* Score	Peak coordinate (x, y, z)
		Right Cerebellum	Pyramis		1	5.12	20 -68 -40
		Right Cerebrum	Putamen		1	5.11	20 10 -14
		Right Cerebrum	Inferior Temporal Gyrus	20	2	5.11	60 -34 -26
		Right Cerebrum	Superior Frontal Gyrus	10	2	5.11	20 70 20
		Right Cerebrum	Caudate		1	5.11	18 10 28
Val/Val PDM	MENS	Left Brainstem	Red Nucleus		6560	62.95	-4 -26 -14
		Right Cerebrum	Postcentral Gyrus	7	285	7.78	16 -54 74
		Left Cerebrum	Insula	13	399	6.96	-36 -32 18
		Left Cerebellum	Cerebellar Tonsil		473	6.80	-4 -42 -48
		Left Cerebrum	Paracentral Lobule	31	60	6.66	-2 -12 48
		Left Cerebrum	Medial Frontal Gyrus		1263	6.63	-8 50 2
		Left Cerebrum	Inferior Temporal Gyrus	20	30	6.57	-40 -14 -48
		Right Cerebrum	Transverse Temporal Gyrus	41	186	6.48	38 -30 10
		Right Cerebellum	Cerebellar Tonsil		113	6.48	30 -34 -44
		Left Cerebrum	Inferior Frontal Gyrus	47	53	6.31	-36 30 -2
		Right Cerebrum	Cuneus	17	37	6.13	6 -100 -2
		Right Cerebrum	Anterior Cingulate	32	50	6.10	18 46 -6
		Left Cerebrum	Paracentral Lobule	5	42	6.08	-10 -46 62
		Right Cerebellum	Declive		42	6.07	22 -60 -24
		Right Cerebrum	Middle Frontal Gyrus	9	91	6.02	36 24 36
		Right Cerebrum	Medial Frontal Gyrus	8	57	5.98	16 28 42
		Left Cerebrum	Middle Frontal Gyrus	11	34	5.92	-26 46 -12
		Left Cerebrum	Middle Occipital Gyrus	19	8	5.88	-52 -74 -12
		Left Cerebellum	Cerebellar Tonsil		26	5.79	-16 -32 -50
		Right Cerebrum	Parahippocampal Gyrus	34	38	5.78	30 4 -16
		Right Cerebellum	Cerebellar Tonsil		6	5.61	16 -58 -58
		Left Cerebrum	Medial Frontal Gyrus	32	10	5.60	-26 30 20
		Right Cerebrum	Middle Temporal Gyrus	21	10	5.59	56 -38 -4
		Right Cerebrum	Cingulate Gyrus	24	13	5.59	22 -16 44
		Right Cerebrum	Inferior Temporal Gyrus	20	1	5.57	50 -24 -38
		Right Cerebrum	Insula	13	5	5.56	38 -44 18

Group	Phase	Laterality	Region	Brodmann area	Cluster size	*t* Score	Peak coordinate (x, y, z)
		Left Cerebrum	Parahippocampal Gyrus	28	8	5.54	-24 -28 -8
		Left Cerebrum	Middle Occipital Gyrus	19	1	5.53	-38 -70 8
		Right Cerebrum	Medial Frontal Gyrus	6	5	5.51	2 -8 68
		Right Cerebrum	Uncus	20	17	5.49	30 -8 -40
		Left Cerebellum	Culmen		5	5.48	-24 -28 -36
		Left Cerebrum	Superior Frontal Gyrus	6	2	5.48	-16 -8 74
		Left Cerebrum	Medial Frontal Gyrus	6	10	5.48	-8 -14 76
		Left Cerebrum	Superior Temporal Gyrus	38	48	5.47	-42 4 -16
		Right Cerebellum	Cerebellar Tonsil		5	5.43	22 -32 -48
		Right Cerebrum	Precentral Gyrus	6	2	5.43	36 -4 30
		Right Cerebrum	Caudate		3	5.40	18 18 14
		Right Cerebrum	Fusiform Gyrus	20	2	5.36	64 -16 -34
		Right Cerebellum	Uvula		8	5.35	6 -68 -38
		Left Cerebrum	Medial Frontal Gyrus	11	9	5.34	-4 32 -14
		Left Cerebrum	Uncus	36	1	5.34	-16 -4 -38
		Left Cerebrum	Caudate		7	5.34	-16 18 10
		Left Cerebrum	Medial Frontal Gyrus	9	3	5.32	-14 38 38
		Left Cerebrum	Superior Temporal Gyrus	38	8	5.31	-44 20 -18
		Left Cerebellum	Inferior Semi-Lunar Lobule		6	5.30	-2 -66 -48
		Right Cerebrum	Inferior Temporal Gyrus	20	1	5.30	54 -32 -24
		Left Cerebrum	Fusiform Gyrus	19	1	5.30	-52 -70 -22
		Left Cerebellum	Inferior Semi-Lunar Lobule		2	5.30	-18 -62 -60
		Right Cerebrum	Cingulate Gyrus	24	1	5.30	22 -16 36
		Left Cerebrum	Extra-Nuclear	13	2	5.27	-40 12 -14
		Left Cerebrum	Claustrum		1	5.27	-34 -4 -10
		Left Cerebellum	Declive		2	5.26	-18 -68 -18
		Left Cerebrum	Caudate		3	5.26	-10 -8 26
		Right Cerebrum	Superior Parietal Lobule	7	1	5.22	20 -70 66
		Left Cerebrum	Paracentral Lobule	5	1	5.22	-18 -44 50
		Right Cerebrum	Caudate		1	5.21	22 -42 8
		Left Cerebellum	Declive		1	5.21	0 -60 -20
		Left Cerebrum	Extra-Nuclear	13	1	5.19	-38 12 -10

Group	Phase	Laterality	Region	Brodmann area	Cluster size	*t* Score	Peak coordinate (x, y, z)
		Right Cerebrum	Medial Frontal Gyrus	10	1	5.19	12 44 -12
		Left Cerebrum	Thalamus		2	5.18	-14 -10 16
		Left Cerebellum	Declive		1	5.18	-52 -70 -28
		Right Cerebrum	Superior Temporal Gyrus	38	1	5.18	44 24 -20
		Right Cerebrum	Precentral Gyrus	6	1	5.17	36 -14 34
		Right Cerebrum	Insula	13	1	5.16	34 -28 24
		Left Cerebrum	Postcentral Gyrus	3	2	5.15	-22 -32 66
		Right Cerebellum	Cerebellar Tonsil		1	5.15	20 -34 -42
		Left Cerebellum	Cerebellar Tonsil		1	5.14	-4 -58 -44
		Right Cerebrum	Cingulate Gyrus	24	1	5.14	8 -4 46
		Left Cerebellum	Cerebellar Tonsil		1	5.13	-8 -58 -44
Val/Met PDM	POV	Left Brainstem	Red Nucleus		4726	52.24	-4 -26 -14
		Right Cerebrum	Middle Frontal Gyrus	6	460	7.89	20 -6 64
		Right Cerebrum	Superior Frontal Gyrus	6	291	7.68	2 16 66
		Right Cerebrum	Inferior Parietal Lobule	40	192	7.31	58 -40 28
		Left Cerebrum	Cingulate Gyrus	32	329	6.81	-14 16 40
		Right Cerebrum	Inferior Frontal Gyrus	47	76	6.74	48 22 -10
		Left Cerebrum	Precentral Gyrus	6	41	6.52	-30 -2 28
		Right Cerebrum	Superior Temporal Gyrus	22	54	6.41	38 -54 12
		Right Cerebrum	Uncus	34	83	6.39	18 -4 -26
		Left Cerebrum	Superior Temporal Gyrus	22	63	6.33	-62 -40 12
		Right Cerebrum	Insula	13	188	6.23	36 22 14
		Right Cerebrum	Caudate		53	6.21	10 18 10
		Left Cerebrum	Medial Frontal Gyrus	10	52	6.21	-6 72 6
		Left Cerebrum	Inferior Parietal Lobule	40	48	6.20	-62 -44 26
		Left Cerebrum	Caudate		64	6.11	-6 10 18
		Right Cerebrum	Medial Frontal Gyrus	32	41	6.09	8 6 48
		Right Cerebrum	Inferior Frontal Gyrus	47	49	6.06	30 26 -6
		Left Cerebrum	Insula	13	29	6.03	-34 -40 26
		Left Cerebellum	Inferior Semi-Lunar Lobule		128	5.95	-2 -60 -54
		Left Cerebrum	Insula	13	75	5.91	-34 -8 18

Group	Phase	Laterality	Region	Brodmann area	Cluster size	*t* Score	Peak coordinate (x, y, z)
		Right Cerebrum	Superior Frontal Gyrus	10	65	5.90	16 64 32
		Right Cerebellum	Culmen		50	5.89	26 -36 -36
		Right Cerebellum	Cerebellar Tonsil		41	5.88	4 -50 -42
		Left Cerebrum	Anterior Cingulate	32	70	5.79	-8 32 -8
		Left Cerebrum	Inferior Frontal Gyrus	47	19	5.78	-30 22 -24
		Left Cerebrum	Uncus	34	29	5.77	-10 -2 -32
		Left Cerebellum	Inferior Semi-Lunar Lobule		32	5.76	-14 -68 -52
		Right Cerebrum	Superior Frontal Gyrus	10	34	5.70	36 56 28
		Left Cerebellum	Cerebellar Tonsil		33	5.66	-4 -42 -54
		Right Cerebrum	Middle Frontal Gyrus	11	4	5.65	26 38 -4
		Right Cerebellum	Uvula		12	5.64	8 -82 -50
		Right Cerebrum	Caudate		32	5.63	20 -36 14
		Right Cerebrum	Superior Frontal Gyrus	10	25	5.62	22 68 2
		Right Cerebrum	Inferior Frontal Gyrus	13	30	5.62	30 10 -12
		Right Cerebrum	Insula	13	3	5.62	40 -20 -8
		Left Cerebrum	Middle Frontal Gyrus	10	36	5.56	-40 58 16
		Left Cerebellum	Cerebellar Tonsil		21	5.56	-8 -34 -44
		Right Cerebrum	Caudate		19	5.55	14 -12 26
		Right Cerebrum	Middle Frontal Gyrus	8	7	5.53	38 22 50
		Right Cerebrum	Cingulate Gyrus	32	5	5.52	14 10 40
		Right Cerebrum	Inferior Frontal Gyrus	47	10	5.52	38 20 -20
		Left Cerebrum	Inferior Parietal Lobule	40	16	5.51	-36 -44 46
		Right Cerebrum	Lingual Gyrus	19	5	5.49	34 -72 0
		Right Cerebrum	Anterior Cingulate	24	17	5.49	10 24 24
		Left Cerebellum	Cerebellar Tonsil		13	5.47	-48 -56 -46
		Left Cerebrum	Cingulate Gyrus	24	15	5.45	-2 -8 44
		Right Cerebrum	Parahippocampal Gyrus	35	7	5.45	18 -14 -32
		Left Cerebrum	Inferior Frontal Gyrus	13	29	5.45	-40 24 8
		Left Cerebrum	Superior Frontal Gyrus	6	1	5.44	-14 12 68
		Right Cerebrum	Precentral Gyrus	6	3	5.43	32 -2 30
		Left Cerebrum	Superior Frontal Gyrus	6	8	5.42	-22 22 64
		Right Cerebrum	Cingulate Gyrus	24	3	5.42	16 -20 48

Group	Phase	Laterality	Region	Brodmann area	Cluster size	*t* Score	Peak coordinate (x, y, z)
		Right Cerebrum	Lingual Gyrus	17	4	5.40	14 -98 -18
		Right Cerebrum	Superior Frontal Gyrus	6	1	5.39	14 -16 70
		Right Cerebrum	Medial Frontal Gyrus	9	2	5.39	10 42 18
		Right Cerebrum	Insula	13	6	5.38	36 -10 24
		Right Cerebrum	Medial Frontal Gyrus	6	4	5.37	4 -24 56
		Left Cerebrum	Middle Occipital Gyrus	18	2	5.36	-32 -100 -4
		Right Cerebrum	Middle Occipital Gyrus	18	7	5.34	38 -94 -2
		Left Cerebrum	Posterior Cingulate	31	1	5.34	-28 -66 18
		Right Cerebellum	Pyramis		1	5.33	52 -70 -42
		Left Cerebrum	Middle Occipital Gyrus	19	5	5.32	-50 -76 -16
		Right Cerebrum	Cingulate Gyrus	32	2	5.32	14 6 44
		Left Cerebrum	Caudate		2	5.31	-28 -44 16
		Right Cerebrum	Inferior Parietal Lobule	40	5	5.31	46 -50 44
		Left Cerebrum	Inferior Temporal Gyrus	20	2	5.30	-34 -14 -50
		Left Cerebrum	Precuneus	7	15	5.30	0 -72 52
		Left Cerebellum	Culmen		13	5.29	-36 -50 -28
		Right Cerebrum	Middle Frontal Gyrus	10	4	5.26	38 44 24
		Right Cerebrum	Middle Frontal Gyrus	10	3	5.26	40 62 8
		Left Cerebrum	Rectal Gyrus	11	2	5.26	-12 12 -32
		Right Cerebrum	Uncus	20	1	5.25	36 -20 -44
		Right Cerebrum	Medial Frontal Gyrus	6	2	5.25	6 -6 66
		Right Cerebellum	Inferior Semi-Lunar Lobule		1	5.25	46 -74 -48
		Left Cerebrum	Superior Frontal Gyrus	6	5	5.24	-22 0 74
		Right Cerebrum	Inferior Parietal Lobule	40	3	5.24	48 -42 40
		Right Cerebrum	Cingulate Gyrus	32	5	5.24	8 14 36
		Right Cerebrum	Superior Parietal Lobule	7	1	5.24	32 -56 66
		Right Cerebrum	Uncus	28	3	5.23	20 12 -32
		Left Cerebellum	Declive		3	5.22	-6 -62 -30
		Right Cerebrum	Postcentral Gyrus	5	1	5.20	34 -48 58
		Right Cerebrum	Parahippocampal Gyrus	34	1	5.18	30 6 -20
		Left Cerebrum	Superior Frontal Gyrus	6	1	5.18	-14 8 68
		Left Cerebellum	Culmen		1	5.18	-34 -48 -22

Group	Phase	Laterality	Region	Brodmann area	Cluster size	*t* Score	Peak coordinate (x, y, z)
		Right Cerebrum	Superior Parietal Lobule	7	1	5.17	36 -52 68
		Left Cerebrum	Thalamus		1	5.17	-12 -12 8
		Right Cerebrum	Cingulate Gyrus	24	1	5.16	12 14 30
		Right Cerebellum	Cerebellar Tonsil		2	5.16	12 -52 -54
		Right Cerebrum	Paracentral Lobule	5	1	5.16	24 -44 46
		Right Cerebrum	Cingulate Gyrus	24	1	5.14	14 6 36
		Right Cerebellum	Cerebellar Tonsil		1	5.14	6 -58 -48
		Right Cerebrum	Middle Frontal Gyrus	6	1	5.13	26 -4 42
		Left Cerebellum	Fastigium		1	5.13	-10 -58 -26
		Left Cerebrum	Thalamus		2	5.13	-8 -16 4
		Left Cerebellum	Pyramis		2	5.13	-34 -84 -44
		Right Cerebellum	Tuber		3	5.13	50 -62 -36
		Left Cerebrum	Middle Occipital Gyrus	18	1	5.12	-34 -98 -2
		Right Cerebellum	Culmen		1	5.12	36 -50 -34
Val/Met PDM	MENS	Left Brainstem	Red Nucleus		18526	60.48	-4 -26 -14
		Left Cerebrum	Middle Temporal Gyrus	21	86	6.49	-46 8 -36
		Right Cerebrum	Cingulate Gyrus	32	170	6.21	8 24 34
		Left Cerebrum	Precentral Gyrus	4	45	6.15	-22 -24 46
		Left Cerebrum	Inferior Parietal Lobule	40	41	6.15	-50 -42 38
		Left Cerebrum	Middle Frontal Gyrus	8	17	6.01	-42 34 44
		Left Cerebellum	Cerebellar Tonsil		29	5.92	-52 -50 -36
		Left Cerebrum	Inferior Frontal Gyrus	45	16	5.75	-34 30 8
		Left Cerebrum	Angular Gyrus	39	9	5.74	-32 -58 32
		Left Cerebrum	Inferior Temporal Gyrus	20	85	5.68	-32 -6 -40
		Left Cerebrum	Inferior Frontal Gyrus	46	39	5.67	-46 42 8
		Right Cerebellum	Culmen		19	5.65	42 -30 -32
		Right Cerebrum	Precuneus	7	4	5.54	18 -54 48
		Left Cerebellum	Tuber		60	5.53	-36 -70 -30
		Left Cerebellum	Cerebellar Tonsil		12	5.53	-48 -58 -40
		Right Cerebrum	Superior Temporal Gyrus	38	6	5.51	22 6 -32
		Left Cerebrum	Medial Frontal Gyrus	6	4	5.46	-16 -12 48

Group	Phase	Laterality	Region	Brodmann area	Cluster size	*t* Score	Peak coordinate (x, y, z)
		Right Cerebrum	Inferior Frontal Gyrus	47	7	5.46	28 16 -10
		Left Cerebrum	Fusiform Gyrus	19	1	5.45	-46 -70 -8
		Right Cerebrum	Middle Temporal Gyrus	38	4	5.43	46 2 -42
		Right Cerebrum	Uncus	20	27	5.43	32 -10 -28
		Left Cerebrum	Postcentral Gyrus	3	1	5.40	-30 -34 48
		Left Cerebrum	Middle Occipital Gyrus	37	2	5.39	-38 -66 -2
		Left Cerebrum	Cingulate Gyrus	32	17	5.39	-10 18 28
		Left Cerebrum	Precuneus	7	3	5.35	-22 -48 48
		Right Cerebrum	Inferior Temporal Gyrus	20	1	5.35	44 -2 -38
		Right Cerebrum	Middle Temporal Gyrus	21	2	5.35	68 -50 -4
		Left Cerebrum	Medial Frontal Gyrus	6	5	5.34	-2 34 36
		Left Cerebrum	Middle Frontal Gyrus	8	2	5.34	-48 26 44
		Right Cerebrum	Superior Frontal Gyrus	10	1	5.33	10 64 -8
		Left Cerebrum	Insula	13	6	5.33	-30 24 0
		Left Cerebrum	Fusiform Gyrus	37	2	5.33	-46 -38 -10
		Right Cerebrum	Superior Temporal Gyrus	38	3	5.30	30 22 -26
		Right Cerebrum	Inferior Frontal Gyrus	47	3	5.29	38 16 -8
		Left Cerebrum	Inferior Temporal Gyrus	20	2	5.29	-46 -8 -18
		Right Cerebrum	Fusiform Gyrus	19	4	5.28	50 -68 -12
		Left Cerebrum	Insula	13	3	5.28	-32 -10 18
		Left Cerebrum	Middle Frontal Gyrus	6	2	5.27	-54 8 42
		Left Cerebrum	Superior Frontal Gyrus	8	3	5.27	-20 50 46
		Right Cerebrum	Precuneus	31	2	5.25	32 -70 14
		Right Cerebrum	Fusiform Gyrus	20	1	5.23	54 -28 -28
		Left Cerebrum	Lentiform Nucleus		1	5.22	-12 6 0
		Right Cerebrum	Middle Frontal Gyrus	6	1	5.21	36 22 60
		Right Cerebrum	Medial Frontal Gyrus	10	1	5.21	14 64 -6
		Right Cerebrum	Superior Temporal Gyrus	13	1	5.21	40 -46 14
		Left Cerebrum	Fusiform Gyrus	20	1	5.21	-44 -6 -22
		Right Cerebrum	Superior Temporal Gyrus	13	1	5.20	40 -48 18
		Right Cerebrum	Superior Temporal Gyrus	22	1	5.18	54 -46 12
		Left Cerebrum	Superior Temporal Gyrus	22	1	5.17	-40 -22 -10

Group	Phase	Laterality	Region	Brodmann area	Cluster size	*t* Score	Peak coordinate (x, y, z)
		Left Cerebrum	Middle Occipital Gyrus	19	1	5.17	-48 -70 6
		Right Cerebrum	Insula		2	5.17	30 22 0
		Right Cerebellum	Culmen		1	5.16	32 -32 -20
		Left Cerebrum	Fusiform Gyrus	37	1	5.16	-36 -46 -14
		Right Cerebellum	Inferior Semi-Lunar Lobule		1	5.16	28 -80 -40
		Right Cerebrum	Uncus	36	1	5.15	24 2 -34
		Right Cerebrum	Sub-Gyral	37	1	5.15	52 -42 -4
		Right Cerebrum	Precuneus	7	2	5.15	22 -50 40
		Left Cerebrum	Superior Parietal Lobule	7	1	5.14	-26 -72 60
		Left Cerebrum	Hippocampus		2	5.14	-36 -10 -16
		Left Cerebrum	Superior Temporal Gyrus	39	1	5.13	-32 -54 28
		Right Cerebellum	Tuber		2	5.13	48 -78 -30
		Right Cerebrum	Fusiform Gyrus	19	1	5.13	52 -72 -14
Met/Met PDM	POV	Left Brainstem	Red Nucleus		11691	40.11	-4 -26 -14
		Left Cerebrum	Uncus	28	386	8.02	-24 6 -28
		Right Cerebrum	Fusiform Gyrus	20	43	6.51	40 -32 -24
		Left Cerebrum	Lingual Gyrus	18	116	6.41	-6 -76 -12
		Left Cerebrum	Superior Frontal Gyrus	6	14	6.16	-12 2 78
		Right Cerebrum	Superior Frontal Gyrus	6	34	6.07	10 4 78
		Left Cerebrum	Insula	13	25	6.05	-30 20 16
		Right Cerebrum	Uncus	28	38	5.96	18 4 -34
		Left Cerebrum	Middle Temporal Gyrus	38	8	5.84	-42 6 -48
		Left Cerebrum	Insula	13	57	5.81	-32 -24 24
		Right Cerebrum	Superior Frontal Gyrus	6	20	5.80	12 20 70
		Right Cerebellum	Pyramis		20	5.69	12 -76 -40
		Left Cerebellum	Culmen		7	5.62	-48 -46 -32
		Left Cerebrum	Postcentral Gyrus	5	8	5.62	-4 -48 74
		Left Cerebrum	Inferior Frontal Gyrus	11	4	5.55	-14 40 -18
		Right Cerebellum	Declive		15	5.54	38 -62 -22
		Right Cerebrum	Middle Temporal Gyrus	38	2	5.48	38 12 -44
		Left Cerebrum	Middle Frontal Gyrus	8	6	5.47	-40 26 52

Group	Phase	Laterality	Region	Brodmann area	Cluster size	*t* Score	Peak coordinate (x, y, z)
		Right Cerebrum	Cingulate Gyrus	24	4	5.45	10 -2 32
		Right Cerebrum	Posterior Cingulate	23	10	5.45	4 -38 22
		Left Cerebrum	Cingulate Gyrus	24	2	5.43	-18 -2 38
		Right Cerebrum	Uncus	38	5	5.42	20 -2 -48
		Left Cerebrum	Caudate		5	5.39	-14 26 -4
		Left Cerebellum	Declive		13	5.34	-28 -64 -20
		Left Cerebellum	Declive		9	5.34	-40 -82 -28
		Left Cerebrum	Parahippocampal Gyrus	36	6	5.33	-26 -44 -14
		Right Cerebrum	Caudate		5	5.29	12 14 16
		Left Cerebellum	Culmen		1	5.28	-12 -68 -14
		Right Cerebrum	Lingual Gyrus	18	9	5.27	16 -78 -16
		Left Cerebrum	Inferior Temporal Gyrus	20	1	5.26	-48 -16 -46
		Left Cerebrum	Lateral Globus Pallidus		2	5.24	-26 -10 0
		Right Cerebrum	Superior Temporal Gyrus	38	4	5.23	34 4 -18
		Right Cerebrum	Caudate		1	5.19	20 -4 26
		Right Cerebellum	Declive		1	5.18	22 -90 -28
		Left Cerebrum	Cingulate Gyrus	24	1	5.18	-16 -8 40
		Left Cerebrum	Insula	13	1	5.17	-34 24 16
		Left Cerebrum	Superior Frontal Gyrus	8	1	5.16	-42 18 56
		Left Cerebrum	Lingual Gyrus	18	1	5.15	-8 -88 -24
		Left Cerebellum	Declive		1	5.12	-32 -60 -14
		Left Cerebrum	Posterior Cingulate	23	2	5.12	-6 -42 24
		Right Cerebellum	Declive		1	5.11	30 -86 -24
		Right Cerebellum	Culmen		1	5.11	4 -60 -8
Met/Met PDM	MENS	Left Brainstem	Red Nucleus		11604	56.03	-4 -26 -14
		Left Cerebrum	Caudate		223	8.21	-18 -24 26
		Right Cerebrum	Superior Temporal Gyrus	38	15	7.00	28 14 -44
		Left Cerebrum	Caudate		59	6.28	-14 -4 26
		Left Cerebrum	Caudate		58	6.25	-22 -38 12
		Left Cerebrum	Lingual Gyrus	18	220	6.22	-6 -92 -22
		Right Cerebellum	Declive		90	6.19	16 -72 -20

Group	Phase	Laterality	Region	Brodmann area	Cluster size	*t* Score	Peak coordinate (x, y, z)
		Right Cerebrum	Inferior Temporal Gyrus	20	19	6.11	58 -60 -22
		Left Cerebellum	Tuber		20	6.10	-56 -48 -30
		Left Cerebrum	Postcentral Gyrus	3	8	6.06	-20 -34 56
		Right Cerebrum	Inferior Frontal Gyrus	47	9	5.93	30 26 -22
		Right Cerebrum	Medial Frontal Gyrus	10	26	5.88	18 46 16
		Left Cerebrum	Superior Frontal Gyrus	6	10	5.64	-22 18 68
		Right Cerebrum	Caudate		34	5.62	14 -24 28
		Right Cerebrum	Inferior Frontal Gyrus	45	17	5.59	50 26 6
		Left Cerebrum	Superior Frontal Gyrus	6	9	5.57	-10 10 68
		Right Cerebrum	Inferior Parietal Lobule	40	21	5.54	42 -60 48
		Left Cerebrum	Cingulate Gyrus	24	5	5.47	-20 -6 42
		Left Cerebellum	Cerebellar Tonsil		15	5.47	-36 -58 -44
		Right Cerebellum	Declive		1	5.41	50 -74 -28
		Right Cerebellum	Cerebellar Tonsil		5	5.40	42 -50 -54
		Right Cerebrum	Rectal Gyrus	11	9	5.40	6 12 -30
		Left Cerebrum	Cingulate Gyrus	31	12	5.40	-10 -46 40
		Left Cerebrum	Uncus	38	1	5.37	-24 14 -34
		Right Cerebrum	Anterior Cingulate	24	1	5.33	12 32 4
		Left Cerebellum	Inferior Semi-Lunar Lobule		3	5.30	-26 -68 -46
		Left Cerebellum	Uvula		7	5.29	-36 -64 -32
		Left Cerebrum	Thalamus		1	5.27	-24 -26 14
		Right Cerebellum	Declive		1	5.26	12 -84 -32
		Right Cerebrum	Anterior Cingulate	32	1	5.26	12 38 22
		Right Cerebrum	Superior Frontal Gyrus	11	3	5.25	24 42 -22
		Left Cerebrum	Anterior Cingulate	33	1	5.24	0 10 22
		Right Cerebrum	Amygdala		2	5.24	34 -8 -14
		Right Cerebrum	Parahippocampal Gyrus	27	1	5.23	24 -30 -6
		Left Cerebrum	Middle Frontal Gyrus	6	2	5.22	-34 6 66
		Left Cerebrum	Middle Frontal Gyrus	6	1	5.22	-26 18 66
		Right Cerebrum	Caudate		4	5.21	14 6 20
		Left Cerebellum	Culmen		1	5.20	-34 -48 -26
		Right Cerebrum	Caudate		1	5.20	18 18 20

Group	Phase	Laterality	Region	Brodmann area	Cluster size	*t* Score	Peak coordinate (x, y, z)
		Left Cerebrum	Precentral Gyrus	6	1	5.19	-32 -16 64
		Left Cerebrum	Uncus	36	1	5.19	-20 -2 -46
		Left Cerebrum	Lingual Gyrus	18	1	5.18	-32 -70 -8
		Left Cerebrum	Inferior Frontal Gyrus	46	1	5.18	-38 40 6
		Right Cerebrum	Medial Frontal Gyrus	9	1	5.17	14 40 18
		Left Cerebrum	Inferior Frontal Gyrus	47	1	5.16	-20 36 -2
		Left Cerebellum	Culmen		1	5.16	-40 -50 -26
		Right Cerebrum	Postcentral Gyrus	2	1	5.16	56 -30 56
		Left Cerebrum	Putamen		1	5.15	-22 6 18
		Left Cerebrum	Caudate		1	5.14	-8 2 20
		Right Cerebrum	Superior Temporal Gyrus	38	1	5.14	32 18 -38
		Right Cerebrum	Rectal Gyrus	11	1	5.13	2 28 -28
		Right Cerebrum	Middle Frontal Gyrus	11	1	5.13	28 42 -20

Supplementary Figure S1. Regions exhibiting significant resting-state functional connectivity with the PAG for between-group and/or between-genotype comparisons

The significance was thresholded at the uncorrected voxel level *p*=0.005, followed by the FDR-corrected cluster level *p*=0.05. The results are superimposed on the SPM T1 template, and the color bar represents *t*-scores. The warm or cold colors represent increased or decreased connectivity, respectively. All figures adopt neurological orientation (left = left). CON, control; MENS, menstrual; PDM, primary dysmenorrhea; POV, periovulatory; Val, valine; Met, methionine.

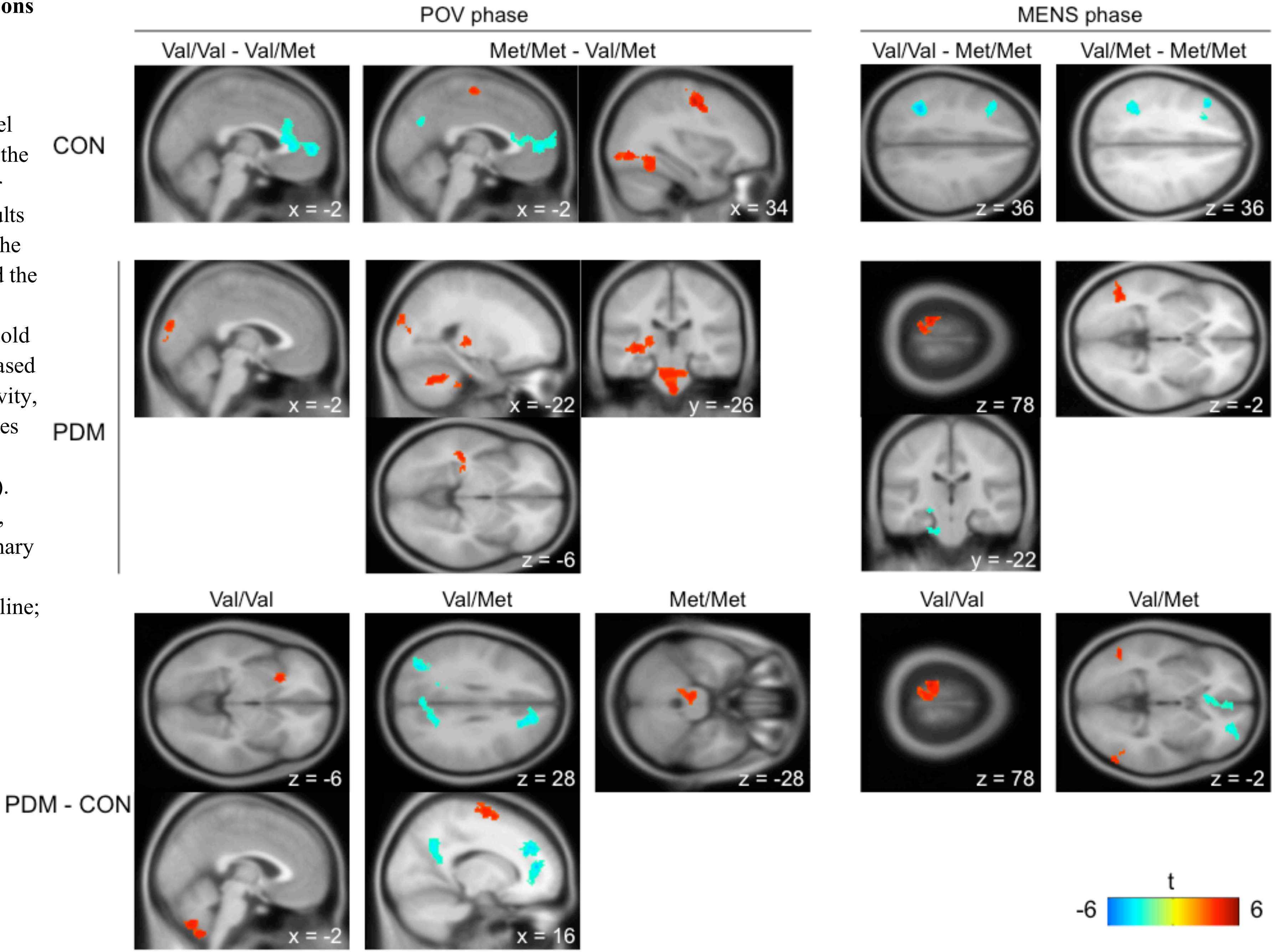

Supplement: Supplementary Information [file srep23639-s1.pdf]
